# Codon bias variation in *Staphylococcus aureus*

**DOI:** 10.1371/journal.pone.0356997

**Published:** 2026-09-09

**Authors:** Kathleen O’Connor, Benjamin D. Mallinger, Nicholas A. Bedard, Robin Patel

**Affiliations:** 1 Division of Clinical Microbiology, Mayo Clinic, Rochester, Minnesota, United States of America; 2 Department of Orthopedic Surgery, Mayo Clinic, Rochester, Minnesota, United States of America; 3 Des Moines University College of Osteopathic Medicine, West Des Moines, Iowa, United States of America; 4 Division of Public Health, Infectious Diseases, and Occupational Medicine, Mayo Clinic, Rochester, Minnesota, United States of America; The Hong Kong Polytechnic University, HONG KONG

## Abstract

**Background:**

*Staphylococcus aureus* causes a multiplicity of human diseases acquired in community and healthcare settings alike around the globe. While most studies focus on coding changes to assess genome evolution and study genetic adaptation, interrogation of silent mutations in the form of synonymous codon usage bias is less well-studied. As such, understanding of patterns in codon bias at the gene and genome levels, and how codon bias impacts protein expression in *S. aureus* remains incomplete.

**Methods:**

The codon bias of 2,565 protein encoding genes from NCTC 8325 was queried against all publicly available closed *S. aureus* genomes. Using public BioSample data, genomes were sorted by disease state, submitting institution, and collection site. Codon bias was assessed at the level of gene and genome using the codon adaptation index (CAI), calculated using 30S and 50S ribosomal genes. Gene set enrichment analysis was applied to determine associations between physiological functions, CAI gene scores, and interquartile ranges. CAI scores were also compared to an *in vitro S. aureus* proteomics database to correlate codon bias and protein expression.

**Results:**

CAI scores varied within and between isolates at the gene and genome levels. Genes with ribosome-associated functions were most enriched among high CAI genes, and had low CAI interquartile ranges (IQR), suggesting selective pressure to maintain high expression of these genes across all *S. aureus* isolates. Genome sequences submitted by Aga Khan University Hospital, Nairobi, Kenya were most different from others. For the LAC USA 300 strain, CAI and protein expression were moderately positively correlated (cor = 0.534, p < 2.2e-16).

**Conclusions:**

Codon bias in *S. aureus* was shown to vary between gene, and to be a source of genetic variation between isolates; CAI and *in vitro* protein expression were positively correlated.

## Introduction

In 2019, *Staphylococcus aureus* was the leading cause of infection-related death in 135 countries, accounting for 1.1 million fatalities [1]. The financial burden of *S. aureus*-related hospitalizations reaches $16 billion yearly in the United States [2]. As *S. aureus* adapts, fitness (defined as the ability to produce offspring and in the process pass a genome to future generations) is optimized based on the environment it encounters. Fitness for a given cell requires a specialized “toolbox” of proteins and secondary metabolites. While changes may occur over the course of a lifespan (phenotypic adaptation/“bet hedging”) [3–18], most occur across generations, as mutations accumulate across genomes (genotypic adaptation) [19–35]. Dynamics of genome evolution therefore play a role in an organism’s survival. Genome evolution does not solely occur due to selective environmental pressures; genome changes also accumulate due to random chance, referred to as “genetic drift” [36], often attributed to “bottleneck”-ing events during infection [37–43]. It is likely that *S. aureus* evolution is driven by both random genetic drift and selective pressure from the various environments it encounters, with sometimes unclear consequences. Bacterial genomic evolution altering protein sequences non-synonymously has been the primary focus of study, potentially obscuring connections between synonymous changes and their impact on bacterial physiology.

Whole genome sequencing has changed the field of bacteriology, with publicly available genomes facilitating research. Initial bacterial Genome Wide Association Studies (GWAS) identified links between *S. aureus* Panton–Valentine leucocidin and pyomyositis [44], for example. While bacterial GWAS connect bacterial genotype and phenotype, more subtle genomic changes may be missed by focusing on coding changes. Codon bias, the unequal rate at which synonymous codons are used in protein coding genes [45,46], is a “silent” genetic adaptation in bacteria, that potentially maximizes fitness [47–52]. Analysis of codon bias could reveal insights into *S. aureus* physiology missed by focusing on coding changes, potentially informing *S. aureus* adaptation.

Codon bias is measured by how codon distributions used in protein-coding genes differ from expected [53–55]. The genetic code is repetitive; multiple codons may encode the same amino acid. Despite this “degeneracy,” codons are not randomly used, with some preferentially used. Depending on optimization of codon usage, favored use may result in variable protein production. The Codon Adaptation Index (CAI) is a metric that uses ribosomal proteins to calculate gene-specific bias [56], with ribosomal proteins assumed to have highly optimized (and biased) codon usage. A gene with a high CAI score, nearing 1.0, is translated at a high rate, leading to high levels of that protein. In contrast, a gene with a low CAI score (~0.1) may not be translated [or may never have been intended to be translated (e.g., tRNAs) and therefore have no codon optimization]. To the best of our knowledge, correlation between CAI and protein expression has not been previously assessed in *S. aureus*. Given its importance as a global pathogen, investigation of all mechanisms the bacterium may have to adapt to the infection environment is relevant. Which genes in *S. aureus* are associated with low or high CAI, whether patterns in CAI score vary between isolates, and whether CAI and protein expression are correlated, are all outstanding unknowns. Additionally, studies exploring gene-specific dynamics typically rely on a single reference genome for each species [57–60], and without addressing within species variations in CAI. A notable exception is a pre-print by Sutradhar et al, which evaluated 72 *Escherichia coli* isolates to link expression with codon bias [61]; the population studied here is about ∼40-fold larger. Additionally, bacterial CAI has primarily been studied at the genomic level – looking at the average CAI of all genes in a genome and correlating this with bacterial physiology [47–50]; here, CAI of individual genes is investigated in addition to trends at the whole genome level.

To globally assess codon bias in *S. aureus*, the CAI of 2,565 protein coding genes from 2,096 publicly available *S. aureus* closed genomes was investigated to evaluate intra-species CAI variation. *S. aureus* was chosen because of its importance to human health, and since it causes several diseases, and can therefore be sourced from a range of environments. Codon bias was explored across individual genes and mean genome CAIs, with gene set enrichment applied to determine predicted physiological impact. Additionally, variation between isolates was assessed by disease state, submitting institution and geographic isolation location at a continent level, with individual genes queried for variation. Finally, a published proteomics dataset was incorporated to correlate CAI with protein expression. Results show that codon bias in *S. aureus* varies between genes, with selection hypothetically driving high CAI scores and with low interquartile ranges for essential genes; codon bias was found to be a source of genetic variation between isolates, likely driven by geography-mediated bottlenecking and genetic drift; lastly, protein expression and CAI were confirmed to be positively correlated using an *in vitro* dataset.

## Results

### Variation in average CAI between genomes

2,872 genes from reference strain *S. aureus* NCTC 8325 were used to extract gene sequences from 6,607 publicly available closed contigs, 4,152 of which were unique *S. aureus* genomes and plasmids from 2,221 *S. aureus* isolates (defined by unique BioSample numbers). When genomes with validated RefSeq accessions were available for a given BioSample, draft genomes were excluded; plasmids were also excluded, leaving 2,223 genomes for contig analysis.

Before further analysis, to ensure all genomes were not biased by GC content due to sequence or assembly methods, the GC content of each genome and the number of genes queried was assessed (data presented in S1 Table). Aside from an outlier which returned 1081 genes (NZ_AP020315.1, KUH140087), all other genomes returned 2,324−2,845 genes. KUH140087 was excluded from downstream analysis, resulting in 2,222 remaining genomes. Mean GC percentage for all genes ranged from 0.331 to 0.335 (data presented in S1 Table). The number of genomes was further decreased to 2,094 to include only those with data available for disease association, institutional collection source, and geographic collection site by continent.

**Fig 1:**
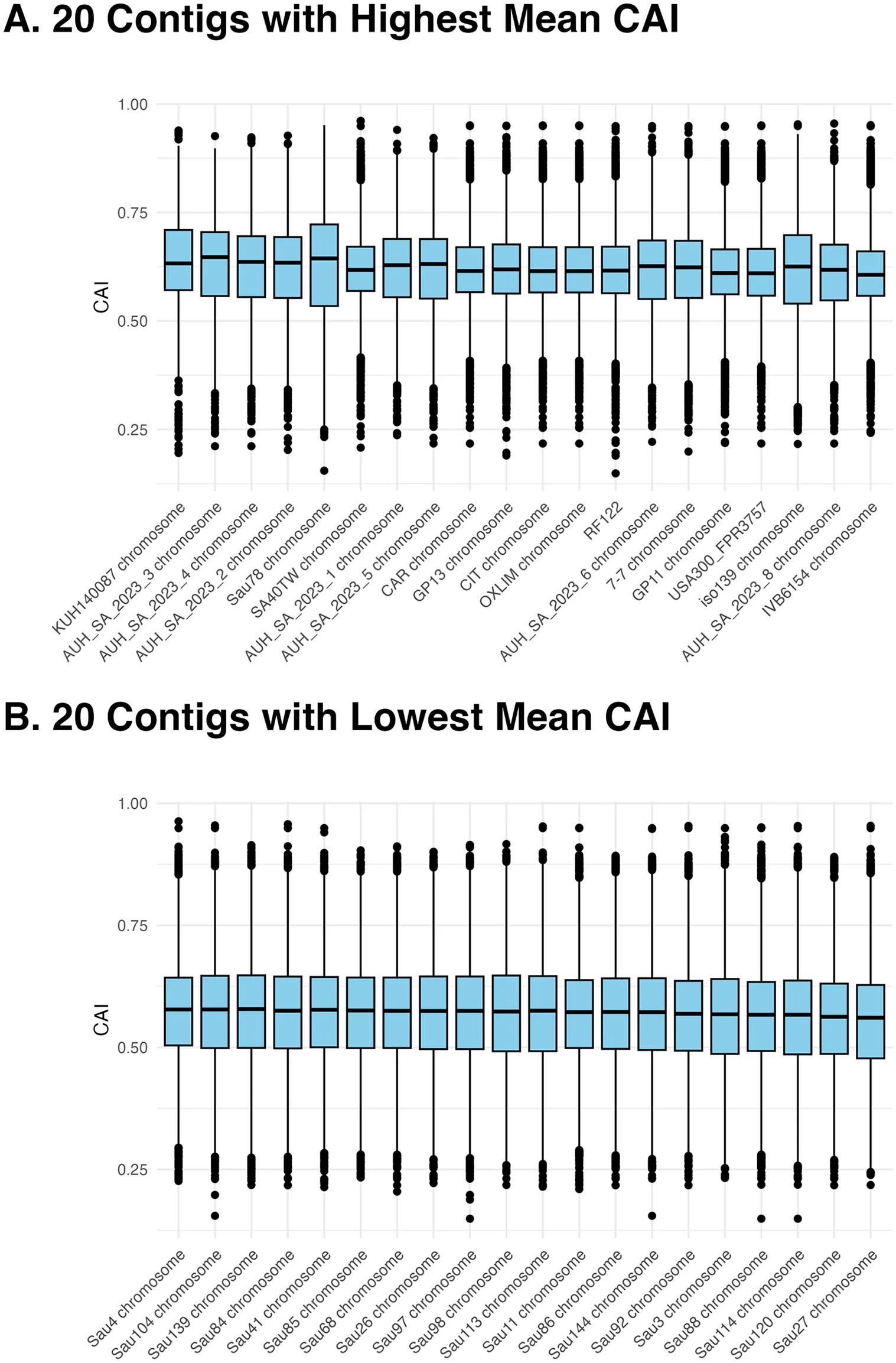
Genome-wide codon adaptation index (CAI) distribution across *S. aureus* isolates. (A) 20 contigs with the highest mean CAI. (B) 20 contigs with the lowest mean CAI. Seven of eight isolate genomes from Aarhus University Hospital were included in the 20 mean highest CAI genomes, as indicated by the “AUH_SA_2023-“ prefix. All 20 isolate genomes with the lowest mean CAI were from Aga Khan University Hospital, as indicated by the “Sau-” prefix. CAI, codon adaptation index.

CAI scores for queried genes per genome were averaged to determine mean genome CAI; tRNA and rRNA genes were excluded, leaving 2,565 protein-encoding genes. The 20 genomes with the highest mean CAI (Fig 1A) and 20 genomes with the lowest mean CAI (Fig 1B) were plotted. Mean CAI for all genes is reported in S2 Table. 7/20 high CAI contigs were from Aarhus University Hospital in Denmark (8 isolate genomes, PRJNA961647), as indicated by the “AUH_SA_2023” prefix, hereafter referred to as AUH [62]. USA300 FRP3757, a methicillin resistant *S. aureus* isolate commonly used as a reference for genomic and laboratory-based studies was also among the 20 genomes with highest mean CAI. The 20 genomes with the lowest mean CAI were from Aga Khan Hospital University in Nairobi Kenya, hereafter abbreviated as AKUHN (82 isolate genomes, PRJNA1046723, as indicated by their “Sau” prefix) [63] These initial findings suggested codon bias adaptations specific to submitting institution, potentially due to regional or disease-specific specific factors.

### Rate of high and low CAI genes by Institutional source

The initial result showing varying codon bias by institutional source was intriguing, so distribution of high and low CAI genes and ribosomal CAI scores by institution, normalized to the total number of genes assessed per institution (AKUHN: n = 2,559; AUH: n = 2,528; Mt. Sinai: n = 2,561; Queen Elizabeth Hospital: n = 2,562; Andrew D. Berti: n = 2,560) was further investigated. Genome sequences submitted by AKUHN had proportionally fewer high-CAI genes (CAI > 0.75) than other institutions: 2.85% (73/2,559) of AKUHN genes had high CAI scores, compared with 4.67% (118/2,528) for AUH, 5.19% (133/2,561) for Mt. Sinai, 5.23% (134/2,562) for Queen Elizabeth Hospital, and 5.43% (139/2,560) for Andrew D. Berti. A test of equality of proportions across the five institutions showed this difference to be statistically significant (χ² = 25.66, df = 4, p = 3.71 × 10 − 5). Because AKUHN appeared to be an outlier, AKUHN was directly compared to the four other institutions pooled together; this confirmed a lower proportion of high-CAI genes in AKUHN (2.85% vs. 5.13%; χ² = 23.34, df = 1, p = 1.36 × 10 − ⁶; 95% CI of the difference: −3.08% to −1.48%). A logistic regression with AKUHN set as the reference level showed that each of the other four institutions had higher odds of a gene being high-CAI relative to AKUHN (Aarhus: β = 0.51, p < 0.001; Berti: β = 0.67, p < 0.001; Mt. Sinai: β = 0.62, p < 0.001; Queen Elizabeth: β = 0.63, p < 0.001), indicating that the deficit of high-CAI genes in AKUHN was consistent across every pairwise comparison.

Genome sequences submitted by AKUHN also had proportionally more low-CAI genes (CAI < 0.5): 11.72% (300/2,559), compared with 4.47% (113/2,528) for AUH, 9.92% (254/2,561) for Mt. Sinai, 9.41% (241/2,562) for Queen Elizabeth Hospital, and 9.84% (252/2,560) for Berti. This difference was also significant (χ² = 91.02, df = 4, p < 2.2 × 10 − ¹⁶), with AKUHN showing the highest proportion of low-CAI genes of any institution.

It was next asked whether the proportion of high-CAI genes that were ribosomal (30S/50S) proteins differed by institution, since ribosomal proteins were used to build CAI reference sets. Ribosomal proteins made up 34.25% (25/73) of high-CAI genes for AKUHN, 21.19% (25/118) for AUH, 30.83% (41/133) for Mt. Sinai, 29.85% (40/134) for Queen Elizabeth Hospital, and 29.50% (41/139) for Berti. A test of equality of proportions found no differences across institutions (χ² = 4.76, df = 4, p = 0.313). A Fisher’s exact test comparing AKUHN against the pooled remaining institutions likewise found no difference (odds ratio = 1.34, 95% CI: 0.76–2.30, p = 0.273), indicating that despite AKUHN’s overall shift toward lower CAI scores, ribosomal proteins were represented among its high-CAI genes at a statistically indistinguishable rate from other sources. Very few ribosomal genes had low CAI scores (<0.5) in any dataset: 0 for AKUHN, Mt. Sinai, and Queen Elizabeth Hospital, 4 for AUH, and 0 for Berti.

Overall, genome sequences submitted by AKUHN had a lower proportion of high-CAI genes and a higher proportion of low-CAI genes than other institutions, while the proportion of high-CAI genes that were ribosomal was statistically similar across institutions.

### Variation in CAI within *S. aureus* by gene

**Fig 2:**
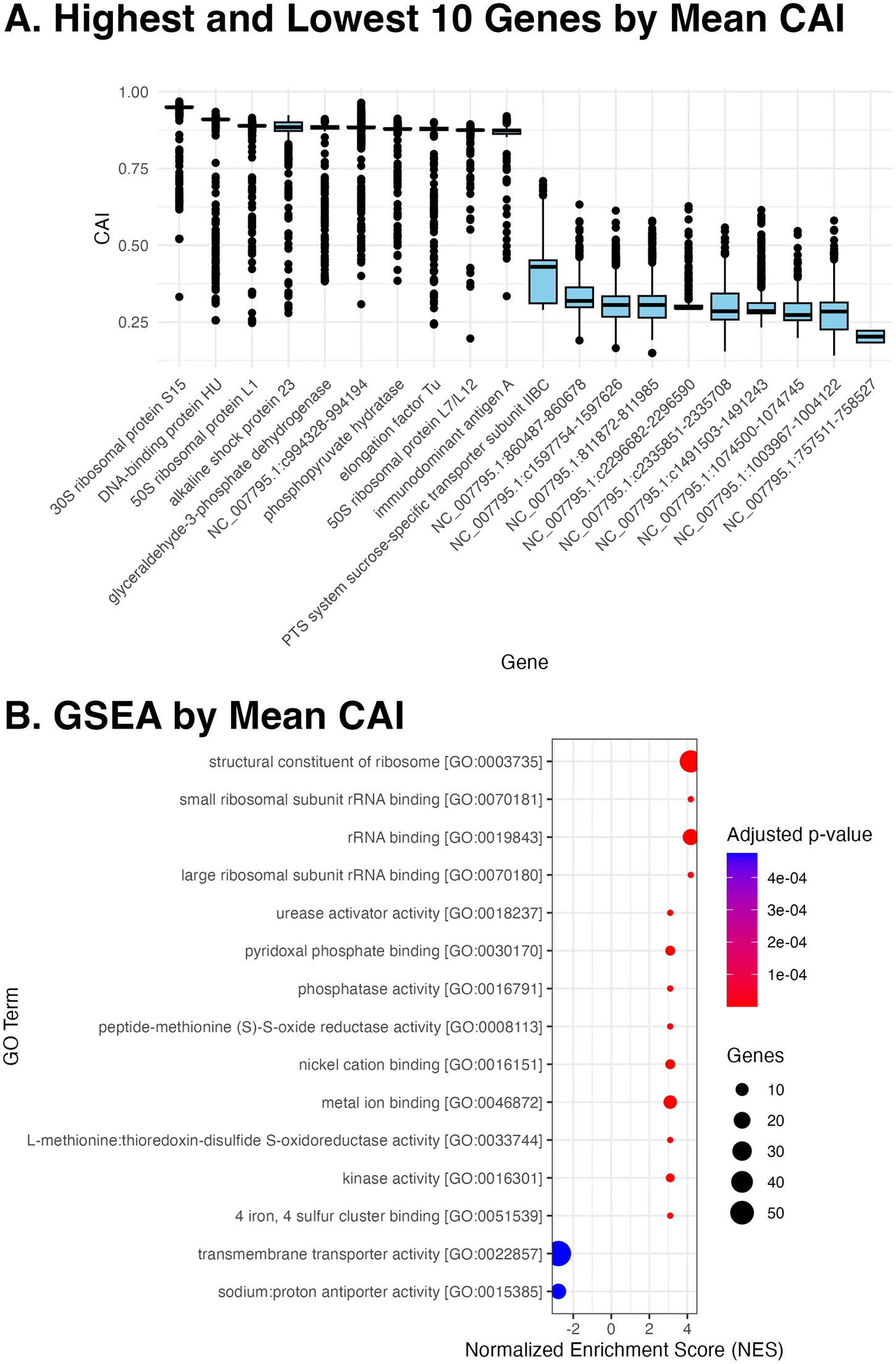
Gene-level codon adaptation index (CAI) and functional enrichment by mean CAI. (A) Boxplots displaying CAI distributions for the 10 genes each with the highest (left) and lowest (right) mean CAI across all isolates. Genes are ordered by decreasing mean CAI. The 10 highest genes have a larger interquartile range (IQR) of mean CAI, while those with the lowest mean CAI genes have a small IQR (explored in more detail in Fig 3). All but one of the lowest CAI genes are hypothetical genes. Genes categorized as “hypothetical proteins” are labeled by NCTC 8325 genetic coordinates. (B) Gene set enrichment analysis (GSEA) dot plot showing GO terms enriched among genes ranked by mean CAI. Point size reflects numbers of annotated genes per GO term; point color reflects Benjamini-Hochberg adjusted p-values. Positive and negative normalized enrichment scores (NES) indicate enrichment among high- and low-CAI genes, respectively. Only GO terms annotated to at least 3 genes with a gene set size between 10 and 300 are shown; the 15 terms with the highest absolute NES are displayed. CAI, codon adaptation index; IQR, interquartile range; GSEA, gene set enrichment analysis; GO, gene ontology; NES, normalized enrichment score**.**

Based on findings that codon bias varied between genomes, variation within each gene was investigated. The 10 genes with highest (left) and lowest (right) mean CAI were plotted (Fig 2A). Multiple genes associated with translation (ribosomal proteins, elongation factor tu) and transcription (DNA-binding protein HU) had high mean CAI. Transcription and translation genes are expected to be universally present and expressed due to their essential functions, suggesting a link between high CAI and total protein expression. Potentially relevant to infection, immunodominant antigen A, universally expressed by *S. aureus,* so much so it can be used as an indicator of sepsis [64–66], was among the genes with high mean CAI. The mean CAI for each gene is presented in S2 Table.

The 10 genes with the lowest mean CAI were all hypothetical genes (listed by NCTC 8325 gene coordinates); they could be untranslated, having no need for optimized codons.

Gene set enrichment analysis (GSEA) was performed on the Gene Ontology (GO) of genes based on CAI score. Significant GO pathways are shown in Fig 2B. Circles to the right and left of the graph are GO pathways with high and low CAI, respectively. Most of the 15 most significant pathways (with 3 or more genes represented) were associated with high CAI scores. These included numerous ribosomal and rRNA related functions including “structural constituent of ribosome,” “small ribosomal subunit rRNA binding,” “rRNA binding,” and “large ribosomal subunit rRNA binding.” Additionally, “urease activator activity,” “pyridoxal phosphate binding,” “phosphatase activity,” “peptide-methionine (S)-S-oxidoreductase activity,” “nickel cation binding,” “metal ion binding,” “L-methionine:thiorexocin-disulfide S-xidoreductase activity,” “kinase activity,” and “4 iron, 4 sulfur cluster binding” were GO pathways with high CAI. There were two GO pathways associated with low CAI scores - “transmembrane transporter activity” and “sodium:proton antiporter activity.”

**Fig 3:**
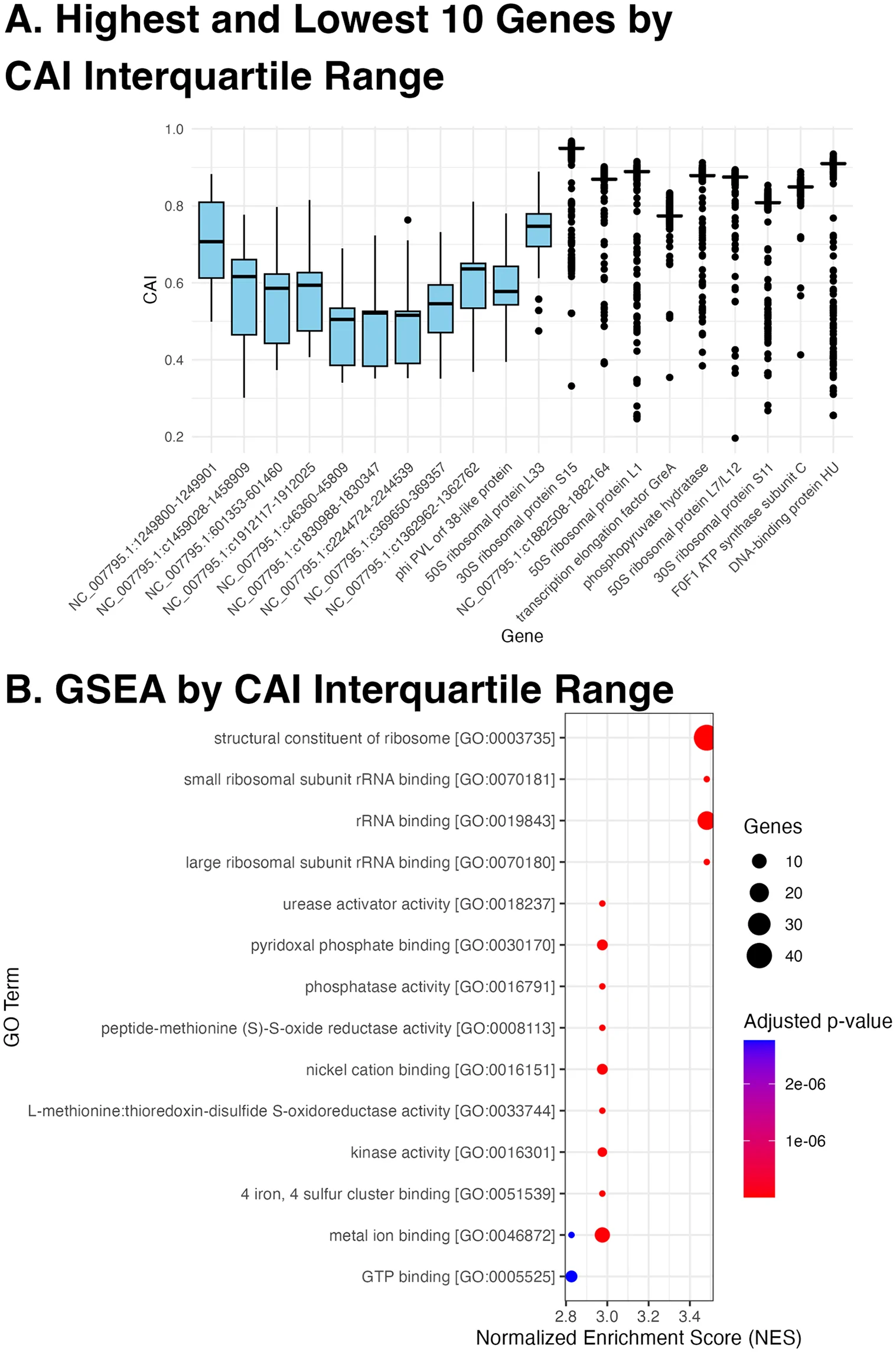
Gene-level codon adaptation index (CAI) variability and functional enrichment by CAI interquartile range. (A) Boxplots displaying CAI distributions for the 10 genes each with the highest (left) and lowest (right) CAI interquartile range (IQR) across isolates. Only genes present in at least 2,000 isolates are included. Genes are ordered by decreasing IQR. (B) GSEA dot plot showing GO terms enriched among genes ranked by CAI IQR. Visualization filters applied as described in Fig 2. CAI, codon adaptation index; IQR, interquartile range; GSEA, gene set enrichment analysis; GO, gene ontology; NES, normalized enrichment score.

There was visual disparity between the range in CAI scores for high and low mean CAI genes, with high CAI genes having low interquartile ranges (IQR) and many low CAI outliers, and low CAI genes having large IQR with few high CAI outliers. Genes with the 10 highest (left) and 10 lowest (right) IQR are shown in Fig 3A; data for all genes is summarized in S3 Table. Genes with low IQR largely coincided with the ten with high mean CAI, and genes with large IQR had lower mean CAI scores (0.552) than the ten with lowest IQR (0.552, 0.841, respectively, Wilcoxon p-value = 0). Most (8/10) high IQR genes were hypothetical proteins, hypothetically reflecting their being untranslated and thus not undergoing consistent selective force driving CAI to be high or low. The exceptions were 2 genes with annotated functions, phi PVL orf 38-like protein and 50S ribosomal protein S15. The former is a protein of unknown function, which appears to be derived from a phage-like particle that carries Panton-Valentine leucocidin genes [67,68]. No information is available on 50S ribosomal protein S15; it could be a mis-annotation of the *S. aureus* 30S ribosomal protein S15 [69].

GSEA was performed using IQR; genes to the right and left of the graph are GO pathways with high and low IQR, respectively (Fig 3B). As with GO pathways associated with high CAI scores, GO functions related to rRNA handling were associated with low IQR, including “structural constituent of ribosome,” “small ribosomal subunit rRNA binding,” “rRNA binding,” and “large ribosomal subunit rRNA binding.” Among the most significant pathways assessed here, no GO pathways associated with high interquartile range were found.

### AKUHN isolates are the most divergent group by CAI

**Fig 4:**
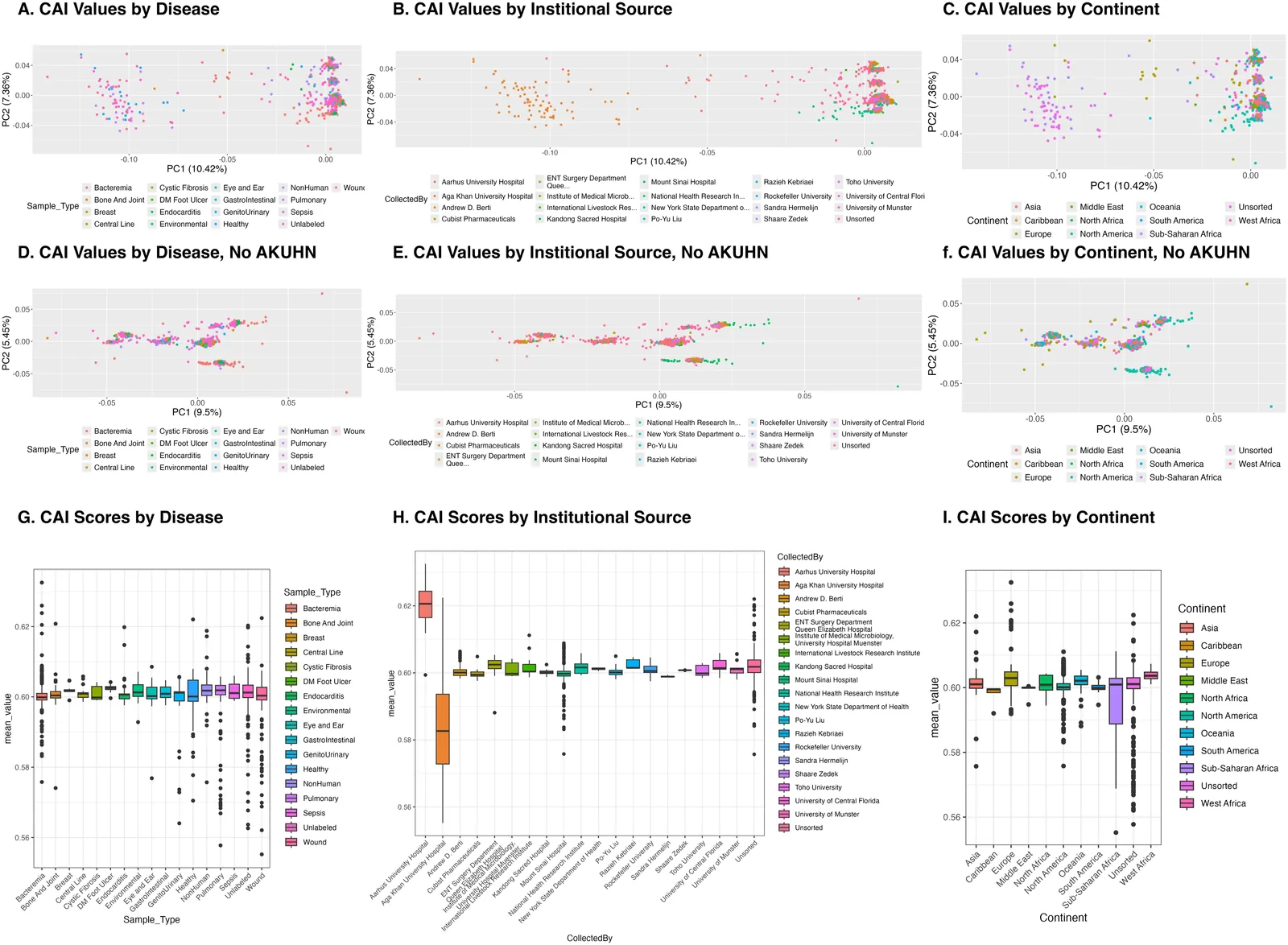
Principal component analysis (PCA) and mean CAI distributions across disease category, institutional source, and geographic region. CAI of 2,565 protein coding genes was plotted; only genomes with a returned query of 2,000 genes were considered. (A) PCA of gene-level CAI values by disease/infection category. (B) PCA by institutional collection source. (C) PCA colored by continental geographic region. (D–F) Equivalent PCA plots with isolates from the Aga Khan University Hospital (AKUHN) excluded. (G) Boxplots of mean per-isolate CAI scores stratified by disease/infection category. (H) Boxplots of mean per-isolate CAI scores stratified by institutional source. (I) Boxplots of mean per-isolate CAI scores stratified by continental geographic region. See S4-S6 Tables for linear matrix model results differentiating between bar plot groups. CAI, codon adaptation index; PCA, principal component analysis.

*Codon Bias by Disease*: Given variation in mean contig CAI within *S. aureus*, potential source of variation was investigated. Using public BioSample data, contigs were sorted by disease state, and CAI scores for queried genes, plotted as principal component analysis (PCA) (Fig 4A).

*Codon Bias by Submitting Institution*: Genomes were next plotted by institutional or research group submitting them to NCBI (Fig 4B, with institutions fewer than 8 isolate genomes binned as ‘Unsorted’). Genomes from AKUHN (peach dots) have the strongest CAI signature, clustering to the left of others, indicating a codon bias signature specific to AKUHN. Aarhus University Hospital genomes cluster between AKUHN and other genomes. The remaining genomes cluster together on component 1 and form four clusters along component 2, although with no apparent separation by submitting institution.

*Codon Bias by Continent*: Genomes were also plotted at the level of continents, as indicated in BioSample data, with genomes without a continent source considered ‘Unsorted’ (Fig 4C). *S. aureus* Sub-Saharan African and many Unsorted genomes clustered to the right of the others, as observed with genome sequences submitted by AKUHN. This indicates that codon bias is impacted more by geographical isolation source than disease state – suggesting bottlenecking and drift rather than host-based selective pressures as drivers of codon bias signature.

Given the strength of AKUHN clustering, AKUHN genomes were removed and data re-plotted (Fig 4D-F).

*Codon Bias by Disease, without AKUHN Genomes:* With genome sequences submitted by AKUHN, clustering appeared largely random, with some possible clustering of bacteremia isolate genomes (Fig 4D, bottom right part of the plot).

*Codon Bias by Submitting Institution, without AKUHN Genomes:* Without genome sequences submitted by AKUHN, clustering did not appear to be driven by submitting institution (Fig 4E), despite observations with AUH genomes in Fig 4B.

*Codon Bias by Continent, without AKUHN Genomes*: Absent genome sequences submitted by AKUHN, clustering appeared random and not driven by geographical source (Fig 4F), suggesting that sequences submitted by AKUHN are different than others.

Generally, when genomes submitted by AKUHN were excluded, genomic clustering by CAI with regards to geography, submitting institution, or disease disappeared.

*CAI by Disease/Infection Category*: Linear mixed modeling revealed minimal variation in CAI across disease and infection categories, with healthy/nasal carriage isolates as the reference group (Fig 4G). Diabetic foot ulcer (estimate = 0.0032, t = 2.26), environmental (estimate = 0.0024, t = 2.52), non-human (estimate = 0.0026, t = 3.14), and unlabeled isolates (estimate = 0.0020, t = 2.64) showed the largest positive deviations from the reference, though all effect sizes were small in absolute terms. No disease category showed a substantial negative deviation from healthy carriage isolates (S5 Table).

*CAI by Collecting Institution*: Institutional source was associated with greater CAI variation than was disease category (Fig 4H). Aarhus University Hospital showed the largest positive deviation from the reference (Mount Sinai Hospital; estimate = 0.0194, t = 13.88), while the Aga Khan University Hospital showed the largest negative deviation (estimate = −0.0160, t = −33.16), consistent with sensitivity analyses performed throughout this study. The University of Central Florida (estimate = 0.0024, t = 4.60), ENT Surgery Department at Queen Elizabeth Hospital (estimate = 0.0026, t = 7.05), and unsorted institution category (estimate = 0.0023, t = 9.32) also showed positive deviations (S6 Table).

*CAI by Continental Geographic Region*: Geographic region was associated with modest CAI variation, with North America as the reference ((Fig 4I). Europe, Oceania and Asia showed positive deviations (estimate = 0.0028, t = 7.81; 0.0017, t = 4.04; and 0.0010, t = 2.48, respectively) and Sub-Saharan Africa the largest negative deviation (estimate = −0.0050, t = −7.52). The Caribbean, Middle East, South America, North Africa, and West Africa showed similar findings to North America (S7 Table).

### Correlation between CAI and proteomics results

**Fig 5:**
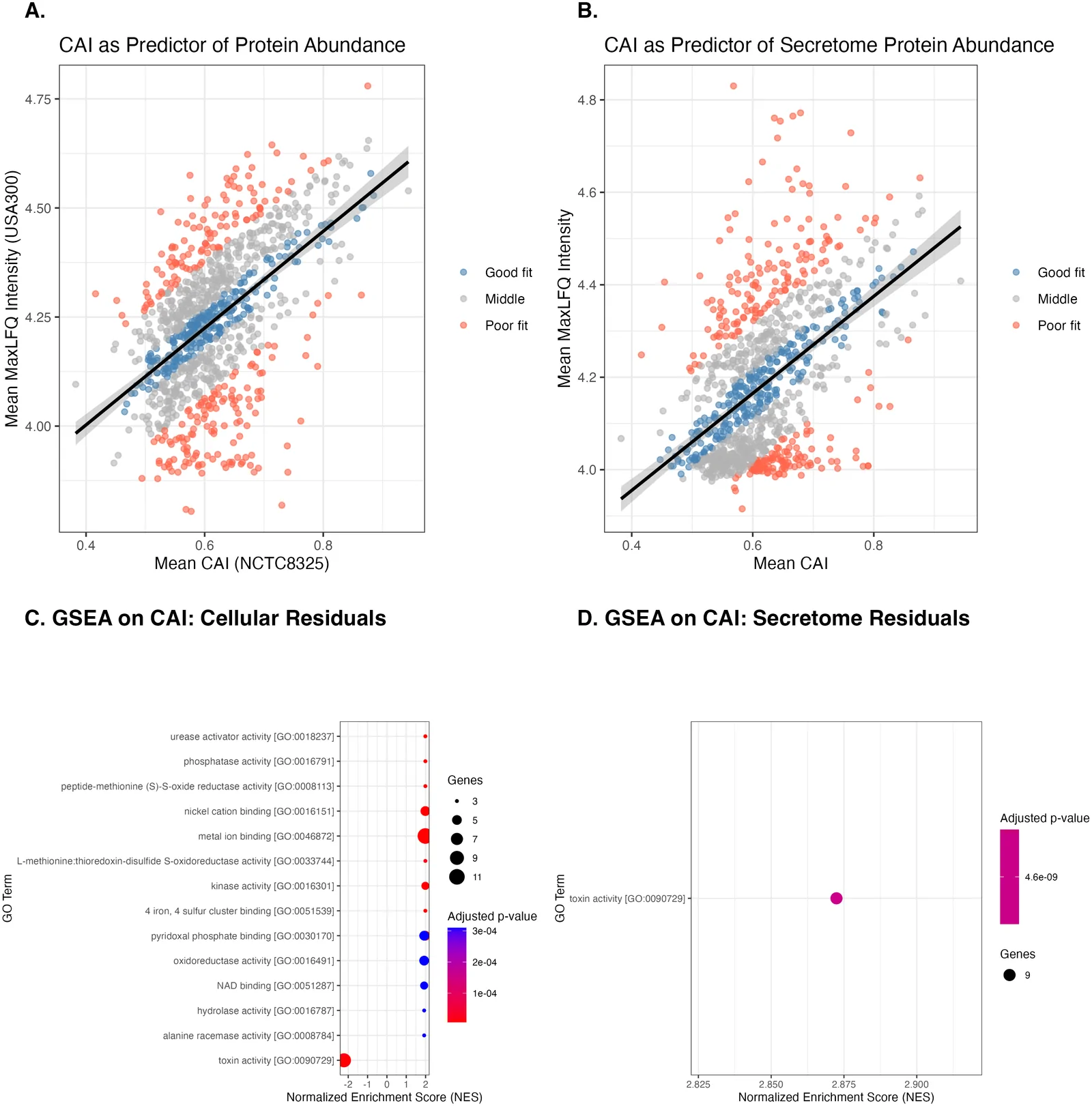
Relationship between codon adaptation index (CAI) and protein abundance in cellular and secreted fractions of *S. aureus.* (A) Scatter plot of mean CAI *versus* mean MaxLFQ intensity for the cellular fraction. Points are colored by goodness of fit to the linear model, with genes in the top quartile of absolute residuals classified as poorly predicted (red) and genes in the bottom quartile classified as well-predicted (blue) by codon bias; Pearson correlation coefficient: 0.534, p-value < 2.2e-16. (B) Equivalent scatter plot for the secreted fraction; Pearson correlation coefficient: 0.513, p-value < 2.2e-16. (C) GSEA dot plot of GO terms enriched among cellular fraction proteins ranked by residual from the CAI-abundance linear model. Positive NES indicates enrichment among proteins more abundant than predicted by codon bias; negative NES indicates enrichment among proteins less abundant than predicted. Visualization filters applied as described in Fig 2. (D) Equivalent GSEA dot plot for the secreted fraction. Proteomics data were obtained from PRIDE Repository PXD053994. CAI, codon adaptation index; GSEA, gene set enrichment analysis; GO, gene ontology; NES, normalized enrichment score; MaxLFQ, maximum label-free quantification..

To assess the physiological impact of CAI scores in *S. aureus*, average CAI scores were correlated with *in vitro* protein expression using a published dataset [70]. Mustor et al. determined the expression of 2,231 proteins in *S. aureus* USA300 LAC in cellular and secreted fractions. The analysis here included genes in NCTC-8325, of which there were 1,010 overlapping genes using the Aureowiki Pangenome code. Only isolates labeled “LAC” were selected to compare LAC proteomics findings with LAC codon bias, not average codon bias of all *S. aureus* isolates. A Pearson’s correlation was run between mean CAI scores and protein expression values from the cellular fraction. The test revealed a correlation of 0.534 (p < 2.2e-16), suggesting a weak positive correlation between CAI and protein expression. To identify genes most and least correlated, the residuals for each gene were determined based on a fit linear model. The least and most correlated genes (>0.75 and <0.25 absolute residual) are plotted in red and blue, respectively. Middle fit genes are plotted in gray (Fig 5A).

The analysis was repeated for the secreted fraction, and Pearson’s correlation determined to be 0.513 (p < 2.2e-16). The secreted fraction protein expression positively correlated with CAI, but was weaker than the protein expression values found in the cellular fraction. CAI was plotted against protein expression from the secreted fraction and labeled as described above (Fig 5B)

All but one significant GO pathway had increased expression in the cellular component compared to what was expected by CAI. These pathways include “urease activator activity,” “phosphatase activity,” “peptide-methione (S)-S-oxide reductase activity,” “nickel cation binding,” “metal cation binding,” “L-methionine-thioredoxin-disulfide S-oxidoreductase activity,” “kinase activity,” “4 iron, 4 sulfur cluster binding,” “pyridoxal phosphate binding, “oxidoreductase activity,” “NAD binding,” “hydrolase activity, and “alanine racemase activity.”

The only pathway with protein expression lower in the cellular fraction than predicted by codon bias was “toxin activity.” Notably, this was the only significant GO pathway present in the secreted fraction, and it was upregulated. This suggests that toxins may be expressed as predicted by codon bias but excreted so as to appear missing when evaluating the cellular fraction.

## Discussion

Codon bias, specifically CAI, was explored across 2,565 protein encoding genes in 2,094 unique closed *S. aureus* genomes to determine potential sources of genetic adaptation relevant to infections in humans. Genes with high CAI scores additionally had low IQR, suggesting selective pressure to maintain certain CAI scores for these genes, even between a highly diverse collection of environments. These genes were most enriched for ribosomal and rRNA binding functions. Genomes submitted by AKUHN were divergent by CAI; divergence is first evident at the whole genome level based on mean genome CAI (Fig 1), and further confirmed using linear mixed models. Using an outside dataset, positive correlation was shown between CAI and protein expression in *S. aureus*, previously only predicted by theory. While CAI variation within and between *S. aureus* genomes is apparent, it is unclear whether selective forces from the environment impact between isolate CAI scores, or if variation is primarily driven by random mutation drift, geographic site of isolation, institution-driven selection biases, clonality, or combinations thereof. This study is a novel first look at the effect of disease, geography and submitting institution on *S. aureus* intra-species CAI variation at a large scale.

The observation that translation and transcription-associated genes — including ribosomal proteins, elongation factor Tu, and DNA-binding protein HU — consistently exhibit the highest mean CAI is consistent with the foundational theory underlying the metric, as these constitutively and abundantly expressed genes are expected to be under strong translational selection. The presence of immunodominant antigen A among high-CAI genes suggests that codon optimization may contribute to maintenance of surface antigen expression across diverse isolates. Genes with the lowest mean CAI are exclusively hypothetical proteins, consistent with relaxed translational selection in minimally or non-expressed genes. GSEA reinforced these findings, with rRNA-related molecular functions, metabolic enzymes involved in core biosynthetic and redox reactions, and metal ion binding pathways enriched among high-CAI genes; enrichment of methionine sulfoxide reductase and thioredoxin-related functions is noteworthy given their roles in oxidative stress defense during host-pathogen interaction. Transmembrane transporter and sodium:proton antiporter activities were associated with low CAI, consistent with their conditionally rather than constitutively expressed nature. IQR analysis further supported these findings, with rRNA-related functions enriched among genes with low CAI variability across isolates, indicating that codon usage in core translational machinery is not only optimized but highly conserved across diverse clinical isolates. No GO pathways were enriched among high IQR genes, likely because highly variable genes were predominantly hypothetical proteins, and thus outside scope of GO annotation.

Linear mixed modeling revealed that while CAI varied modestly across disease or infection type, they were not strong predictors of codon bias, and instead CAI varied most in AKUHN isolates from Sub-Saharan Africa. Greater variation was observed by collecting institution and geographic region, with Aga Khan University Hospital and Sub-Saharan African isolates showing the most pronounced negative deviations. First identified by mean CAI at the level of the genome, genomes from AKUHN had a CAI signature defined by more low CAI genes than those found in isolates from other submitting sources. It is unknown what effects a genome with low CAI genes might have on *S. aureus*, but hypothetically associated isolates might present themselves differently than other *S. aureus* isolates in the laboratory and patients. Supporting this hypothesis, *S. aureus* genomes isolated from sub-Saharan Africa have been reported as divergent from those from Europe and the United States [71–83]. Potentially, for these genomes, a metric other than CAI would better predict codon optimization. Potentially, genetic shift, including the availability of tRNAs, may have altered codon optimization AKUHN genomes’ ribosomes, as tRNAs drive codon bias [84–92]. Future investigations should assess this utilizing alternative codon bias metrics that include tRNA contributions. Findings could possibly signal a speciation or subspeciation event, with the sub-Sahara African isolates forming a novel *Staphylococcus* “pseudo-*aureus*” style species. Speciation in microbes is complex [reviewed by Shapiro et al. [93]]; codon bias may merit consideration as a contributor to speciation. This finding suggests that *S. aureus* CAI intra-specific variation is driven by genetic drift and bottlenecking due to geographic isolation, rather than selection from specific infection environments.

Correlation between CAI and protein expression was assessed using an outside dataset of *in vitro S. aureus* proteomics data. The moderate positive correlation between CAI scores and protein expression in both cellular (Corr = 0.534) and secreted (Corr = 0.513) fractions supports the utility of CAI as a proxy for translational efficiency in *S. aureus*, while also highlighting limitations of codon bias as a sole predictor of protein abundance. Although CAI reflects the degree to which a gene’s codon usage matches the cell’s tRNA pool, imperfect correlation suggests that additional transcriptional and post-translational regulatory mechanisms contribute to final protein levels. Enrichment of most significant GO pathways in the cellular fraction beyond what CAI would predict may reflect translational buffering or increased protein stability for core metabolic functions, such as oxidoreductase and hydrolase activity, that are critical to cellular homeostasis. The singular exception (toxin activity) is noteworthy, as its apparent underrepresentation in the cellular fraction may be reconciled by its strong upregulation in the secreted fraction, consistent with the known biology of *S. aureus* virulence factor secretion [94]. This pattern suggests that codon optimization in toxin-encoding genes may serve to sustain high translational output rapidly directed extracellularly, rather than accumulating intracellularly, and underscores the importance of examining both cellular and secreted proteomes when interpreting codon bias data.

In conclusion, querying the CAI of a large collection of closed *S. aureus* contigs revealed variation in CAI within and between *S. aureus* genomes. Results suggest that codon bias is driven by a combination of selective forces (primarily at the gene level) and random mutational drift (primarily at the whole genome level), lending support to the selection-mutation-drift theory of synonymous codon usage [95]. While this study serves to progress the field of codon bias and *S. aureus* pathogenesis by studying the CAI of many genes across a large *S. aureus* population, there are limitations. First, genes were annotated based on NCTC 8325 sequences, and therefore only capture the its 2,872 annotated genes, and 2,565 protein encoding genes. While this was helpful when comparing CAIs between genomes, non-NCTC 8325 genes were not considered. Beneficially, this study surveyed 2,096 closed genome contigs and 2,565 genes, ensuring that results should be generalizable across *S. aureus*. This study focused on a single bacterial species; others will be necessary to determine if CAI variation between genomes generalizes beyond *S. aureus*. Additionally limiting this study, BioSample data for many submissions was missing or challenging to interpret, hindering classification by geography and disease state. Further studies with detailed data on *S. aureus* physiology are needed to better discern connections between CAI and disease state/clinical outcome. Publicly available metadata may introduce bias, as institutional differences could reflect variation in sequencing pipelines or clonality rather than true biological differences; this as an inherent constraint of retrospective metadata analysis and thus caution is needed when interpreting institution-level comparisons. Notably, bacterial GSEA is still a field in its nascent phase and does not have the same infrastructure as for *Homo sapiens*; the analysis relied on clusterProfiler [96]. Lastly, while results were compared to an *in vitro* dataset, this study did not incorporate *in vivo* experiments and thus does not assess any consequences on host-microbe interactions driven by codon bias; further studies utilizing experimental evolution *in vitro* and (ideally) *in vivo* should address key drivers of codon bias signatures, and determine whether CAI variation in *S. aureus* is clinically impactful.

## Methods

### Sequences

*S. aureus* sequences were downloaded from the NCBI using the command line and rehydrated; on September 23^rd^, 2024, this amounted to 6,607 contigs. Before downstream CAI analysis, contigs were grouped by title, with draft contigs ignored, resulting in 4,152 unique titled entries for analysis [i.e., pt214 plasmid pER05716.3A.5, SCAID OTT1–2022 (150) chromosome].

### Exclusion criteria and data filtering

Duplicate sequence entries were removed prior to analysis, retaining only unique sequence identifiers. To ensure consistency in genomic reference standards, contigs were filtered by accession prefix hierarchy on a per-BioSample basis. For isolates with fully validated RefSeq accessions (prefixed “NC_”), contigs bearing two-letter accession prefixes (e.g., “CP,” “AP”) were excluded. Similarly, where a WGS RefSeq accession (prefixed “NZ_”) was present, non-RefSeq two-letter prefixed contigs were removed. This ensured that only the most established and curated sequence representation was retained for each isolate. Following accession-based filtering, redundant rows were removed to retain unique entries. The reference strain NCTC8325 (accession NC_007795.1) was excluded to avoid circularity, as it served as the genomic coordinate reference for gene annotation.

Genomes were filtered to retain only those with a minimum of 2,000 annotated genes, excluding likely non-*aureus* isolates; this resulted in exclusion of KUH140087. Plasmid sequences and genes annotated as tRNA or ribosomal RNA were removed. For genes with unique annotated descriptions, gene description was used as the identifier; hypothetical proteins were identified by genomic coordinates.

### GC content calculation

Genome-wide GC content was calculated from the same multi-FASTA file used for CAI analysis. Per-gene GC content was computed using the letterFrequency function from the Biostrings package in R [97], and mean GC content averaged across genes within each contig. Contig-level GC values were merged with metadata using the same accession prefix filtering pipeline applied in the CAI analysis, retaining only the most curated sequence representation per BioSample. Plasmid sequences were excluded. Mean and standard deviation of GC content, along with gene count, were retained per contig for downstream analysis.

### Organization of contigs by bioSample ID to determine isolation and disease sources

Accession numbers were extracted from the multifasta file and queried against BLAST for BioSample identifiers (IDs). BioSample IDs were entered into NCBI Batch Entrez in binned groups, 200 IDs at a time; all available BioSample data were downloaded into a txt file. Isolates were parsed by disease state, submitting institution, or geography (continent) in R studio (see Github for code files for detailed parsing instructions). Briefly, they were parsed as follows:

*Disease Type:* Publicly available Biosample metadata was downloaded from NCBI BioSample and processed in R (version 4.3.2) using the tidyverse package. Isolates were categorized into clinically relevant groups based on structured metadata fields including host disease, isolation source, host health state, and related attributes. Categories included bacteremia, bone and joint infection (including periprosthetic joint infection and osteomyelitis), breast infection, central line-associated bloodstream infection, cystic fibrosis, diabetic foot ulcer, endocarditis, gastrointestinal infection, genitourinary infection, healthy/colonization, non-human host, pharyngitis, pneumonia, pulmonary infection, sepsis, sinusitis (with and without nasal polyps), superficial skin infection, wound/soft tissue infection, eye and ear infection, and environmental/food isolates. Duplicate Biosamples were removed prior to categorization. Isolates were assigned to categories using keyword and phrase matching against metadata fields; isolates matching exclusion criteria (e.g., misclassified accessions identified by manual review) were removed from individual categories. A subset of isolates with missing or ambiguous metadata (including those lacking host disease or isolation source annotations) were retained as an unlabeled category. Each Biosample was verified to belong to a single category, with any duplicates across categories identified and resolved.

*Institutional source:* Biosample collection source was extracted from the metadata field for each isolate. Isolates lacking collection source information, or for which this field was annotated as missing, were assigned to an unclassified category. Abbreviated or non-standardized institution names were harmonized to their full institutional names (*e.g.,* “NYS DOH” to “New York State Department of Health,” “NHRI” to “National Health Research Institute”). Institutions represented by fewer than 8 isolates were collapsed into the unclassified category to avoid sparse groupings in downstream analyses.

*Geographical isolation source:* Geographic location was extracted from the geographic location metadata field. Isolates with missing, ambiguous, or non-informative geographic annotations (e.g., “not applicable,” “unknown,” “not collected”) were assigned to an unclassified category. Where geographic entries included sub-national detail (e.g., “Sweden: Stockholm”), only the country-level designation was retained. Non-standardized entries were harmonized to consistent country names (e.g., “USA” to “United States”). Countries were then mapped to broader geographic regions: North America, South America, Caribbean, Europe, Asia, Oceania, North Africa, West Africa, Sub-Saharan Africa, and Middle East. Isolates for which a continental region could not be determined were retained in the unclassified category.

### Annotation of *S. aureus* genes using nctc 8325 genes and gamma

*S. aureus* subspecies *aureus* NCTC 8325 ASM1342v1 (GCF_000013425.1) genes (n = 2,872) were downloaded from NCBI (www.ncbi.nlm.nih.gov/datasets/gene/GCF_000013425.1/) as a multifasta and labeled by NCTC 8325 genome position. GAMMA.py was used to query downloaded NCTC 8325 genes against the 6,607 *S. aureus* contigs, with the -f flag used to extract sequences [98].

### CAI calculation

Codon adaptation index (CAI) was calculated for all genes across each *S. aureus* genome using the cubar package in R.[99] Gene sequences were obtained from a multi-FASTA file generated by aligning query genomes against the *S. aureus* NCTC8325 reference, with duplicate sequence entries removed prior to analysis. Ribosomal genes (30S and 50S ribosomal proteins) were identified for each genome by matching annotated genomic coordinates against gene descriptions obtained from the NCBI Gene dataset for *S. aureus* NCTC8325 (taxon 93061). Ribosomal gene sequences were used as the reference highly expressed gene (HEG) set, as ribosomal proteins are constitutively highly expressed and represent the standard reference for CAI calculation. Codon frequencies were calculated separately for all genes and for ribosomal genes in each genome using count_codons, and relative synonymous codon usage (RSCU) was estimated from the ribosomal gene codon frequencies using est_rscu. CAI values for all genes were then computed using get_cai with the isolate-specific ribosomal RSCU as the reference, such that each genome’s CAI values were normalized to its own codon usage context.

### Gene CAI classification by Institution

For each institution, mean CAI was calculated per gene by averaging CAI values across all contigs assigned to that gene within the institution’s dataset. Genes were classified as high-CAI if their mean CAI exceeded 0.75, and low-CAI if their mean CAI was below 0.5. The number and percentage of high- and low-CAI genes were calculated for each institution relative to the total number of genes assessed for that institution. Genes were classified as ribosomal proteins if their gene name matched “50S ribosomal protein” or “30S ribosomal protein” (case-insensitive). The number and percentage of ribosomal proteins among high- and low-CAI genes were calculated separately for each institution, as was the mean CAI of ribosomal protein genes specifically. Institution-specific gene sets were combined, and a presence/absence matrix constructed indicating whether each gene was detected in each institution’s dataset, to allow qualitative comparison of gene content across institutions.

Normality of mean CAI values was assessed using the Shapiro-Wilk test; because normality was rejected (W = 0.985, p < 0.001), non-parametric methods were used for comparisons of continuous CAI values. Differences in mean CAI across all five institutions were tested using a Kruskal-Wallis rank sum test, followed by pairwise Wilcoxon rank-sum tests with Benjamini-Hochberg correction for multiple comparisons to identify which institution pairs differed significantly.

Differences in the proportion of high-CAI and low-CAI genes across institutions were assessed using a five-sample test of equality of proportions (prop.test). Because AKUHN was identified as a consistent outlier, a two-sample test of equality of proportions was additionally used to compare AKUHN directly against the four other institutions pooled together. To confirm this pattern held across all pairwise institutional comparisons, a logistic regression model was fit with high-CAI gene status (CAI > 0.75) as the binary outcome, institution as a categorical predictor, and AKUHN set as the reference level.

The proportion of high-CAI genes that were ribosomal proteins was compared across institutions using a five-sample test of equality of proportions, and AKUHN was compared directly against the pooled remaining institutions using Fisher’s exact test on the corresponding 2 × 2 contingency table, given the relatively small cell counts involved. All p-values from pairwise comparisons were corrected for multiple testing using the Benjamini-Hochberg method where applicable. A significance threshold of α = 0.05 was used throughout.

### Gene set enrichment analysis

*GSEA by CAI Score:* To identify biological processes enriched among genes with high or low codon adaptation, gene set enrichment analysis (GSEA) was performed using the clusterProfiler package in R [96]. Gene Ontology (GO) annotations for *S. aureus* NCTC8325 were obtained from UniProt and filtered to retain only genes present in the CAI dataset. A ranked gene list was constructed using mean CAI values, with genes ordered from highest to lowest CAI. GSEA was performed against GO terms using this ranked list, with a significance threshold of adjusted p < 0.05. GO term-to-gene mappings were constructed from GO IDs linked to NCTC8325 ordered locus tags, with multiple GO terms per gene expanded into separate rows. Where available, molecular function (MF) annotations were used as GO term labels in place of GO term descriptions; where molecular function annotations were absent, the GO term description was retained.

For visualization, results were filtered to retain GO terms annotated to at least 3 genes in the dataset and with a gene set size between 10 and 300 genes, to exclude both poorly annotated and overly broad terms. The 15 terms with the highest absolute normalized enrichment score (NES) were selected for display. Results were plotted as a dot plot with NES on the x-axis, GO term on the y-axis, point size reflecting the number of annotated genes, and point color reflecting the adjusted p-value.

*GSEA by CAI Range:* A complementary GSEA was performed using the per-gene inter-quartile CAI range across isolates as the ranking metric, rather than mean CAI, to capture genes whose codon usage varied substantially across the dataset. The ranked gene list was constructed by ordering genes from highest to lowest CAI range, with GO annotation filtering, term-to-gene mapping, and significance thresholds applied as described above. Visualization filters were likewise identical, retaining GO terms annotated to at least 3 genes and with gene set sizes between 10 and 300, with the 15 terms of highest absolute NES selected for display.

### Generation of PCA plots

To examine population structure across isolates, CAI values were first averaged across duplicate genome position entries within each Biosample, then reshaped into a wide-format matrix in which rows represented individual Biosamples and columns genomic positions. Missing CAI values were imputed using the column mean prior to analysis. PCA was performed on the resulting matrix using the prcomp function in R with feature scaling (scale. = TRUE). PCA was visualized using the ggfortify package, with isolates colored by disease category, institutional collection source, and continental geographic region in separate plots. To assess the potential influence of a single high-contributing institution, a sensitivity analysis was performed by repeating the PCA after excluding isolates from the Aga Khan University Hospital.

### Linear mixed modeling

Codon Adaptation Index (CAI) values were compared across sample type, continent of origin, and collecting institution (Fig 4G–I). For each grouping variable, an initial one-way ANOVA (aov() in R) was used to test for an overall association between CAI and the grouping variable, treating individual measurements as independent observations.

Because samples were nested within individual patients or specimens (BioSample), a linear mixed-effects model was subsequently fit for each comparison to account for non-independence of repeated measurements from the same BioSample. CAI values were scaled (z-scored) prior to modeling. Linear mixed models were implemented using the *lmer()* function from the lme4 package [100] in R Studio, with the grouping variable (Sample_Type, Continent, or CollectedBy) modeled as a fixed effect and BioSample included as a random intercept to account for within-sample correlation.

For each model, the reference level of the fixed effect was set as follows: “Healthy” for Sample_Type, “North America” for Continent, and “Mount Sinai Hospital” for CollectedBy, allowing all other levels to be interpreted relative to these baselines. Fixed-effect coefficients, standard errors, and associated statistics were extracted using the tidy() function from the broom.mixed package.[101]

Boxplots summarizing mean CAI by group (Fig 4G–I) were generated using ggplot2, with each point representing the mean CAI per BioSample.

### Comparison with external *S. aureus* proteomics dataset

*Normalization:* MaxLFQ intensity values for cellular and secreted protein fractions were obtained from the combined_protein.tsv file deposited in the PRIDE proteomics data repository, PXD053994 [70]. Separate processing pipelines were applied to the cellular and secreted fractions. Missing values were assumed to be missing not at random (MNAR) and imputed using the minimum probability method (MinProb, q = 0.01) as implemented in the DEP package in R. Following imputation, quantile normalization was applied across isolates using the preprocessCore package to reduce between-sample technical variation. Normalized intensity values were then log2-transformed. The final processed matrices, with samples as rows and proteins as columns, were exported separately for the cellular and secreted fractions for downstream analysis. Following normalization, mean log2 MaxLFQ intensity was calculated across all samples for each protein in both the cellular and secreted fractions independently.

*Nomenclature standardization:* To enable comparison with CAI values, proteins were annotated with standardized cross-reference identifiers through a multi-step mapping pipeline. UniProt accession numbers were extracted from protein identifiers and queried against the UniProt REST API to retrieve gene names and corresponding KEGG locus tags for *S. aureus* USA300_FPR3757. USA300_FPR3757 locus tags were then mapped to a pangenome ortholog table (AureoWiki) to obtain pan-genome identifiers shared across strains [68]. Proteins with one-to-many mappings to the pangenome (accessions P0C7B5, P0C7Y0, and P0C817) were excluded from downstream analysis due to ambiguous ortholog assignments.

Mean CAI values were similarly mapped to pangenome identifiers via genomic coordinates. Briefly, CAI values calculated per gene were linked to NCTC8325 locus tags by matching annotated genomic range start and stop positions, then joined to gene-specific annotation data and subsequently to the pangenome ortholog table. Proteins and CAI values were then merged on shared pangenome identifiers.

*Pearson correlation between CAI and proteins:* The relationship between codon bias and protein abundance was assessed for the cellular and secreted fractions by computing the Pearson correlation between mean CAI and mean MaxLFQ intensity across all proteins with available data in both datasets. A linear model was fit to quantify this relationship, and residuals were calculated to identify genes for which CAI was a relatively good or poor predictor of protein abundance.

*Gene Set Enrichment Analysis (GSEA) by Residuals:* To identify biological processes enriched among proteins whose abundance was poorly predicted by codon bias, GSEA was performed using linear model residuals as the ranking metric. As described above, residuals were derived from a linear model regressing mean MaxLFQ intensity against mean CAI, where positive residuals indicate proteins more abundant than predicted by codon bias and negative residuals indicate proteins less abundant than predicted. Genes were ranked from highest to lowest residual and GSEA was performed against GO annotations as described above. This analysis was performed separately for the cellular and secreted fractions. GO annotation filtering, term-to-gene mapping, visualization filters, and plot formatting were applied as described above for the GSEA by CAI Score and Range analyses.

### Data visualization

Graphs were created with ggplot and assembled in R studio.

## Supporting information

S1 Table Genome-wide GC content by isolate. Mean GC content calculated across all chromosomal genes per *Staphylococcus aureus* isolate. Values represent the mean and standard deviation of per-gene GC content within each contig. n_genes indicates the number of annotated genes used to calculate mean GC content for each isolate.
(CSV)

S2 Table Mean codon adaptation index (CAI) per gene across all *Staphylococcus aureus* isolates. Mean CAI calculated across all isolates for each annotated protein-coding gene in *S. aureus* NCTC8325. tRNA and ribosomal RNA genes were excluded. Genes are sorted in descending order of mean CAI. GenomePosition refers to the NCTC8325 genomic coordinates used to identify each gene.
(CSV)

S3 Table Codon adaptation index (CAI) variability per gene across all *Staphylococcus aureus* isolates. Interquartile range (IQR), standard deviation (SD), and quartile values (Q1, Q3) of CAI calculated across all isolates for each annotated protein-coding gene in *S. aureus* NCTC8325. tRNA and ribosomal RNA genes were excluded. Only genes present in at least 2,000 isolates were included. Genes are sorted in descending order of IQR.
(CSV)

S4 Table Linear mixed model results for codon adaptation index (CAI) by infection/disease category. Fixed effect estimates from a linear mixed model assessing the association between infection or disease category and CAI, with BioSample included as a random effect to account for within-isolate correlation. CAI values were scaled prior to modeling. Healthy/nasal carriage isolates were used as the reference category. Estimates represent the difference in scaled CAI relative to the reference group.
(CSV)

S5 Table Linear mixed model results for codon adaptation index (CAI) by continental geographic region. Fixed effect estimates from a linear mixed model assessing the association between continental geographic region and CAI, with BioSample included as a random effect. CAI values were scaled prior to modeling. North America was used as the reference region. Estimates represent the difference in scaled CAI relative to the reference group.
(CSV)

S6 Table Linear mixed model results for codon adaptation index (CAI) by collecting institution. Fixed effect estimates from a linear mixed model assessing the association between collecting institution and CAI, with BioSample included as a random effect. CAI values were scaled prior to modeling. Mount Sinai Hospital was used as the reference institution. Estimates represent the difference in scaled CAI relative to the reference group.
(CSV)
