# Supplementary material for: Codon bias variation in Staphylococcus aureus
Source: PLoS One. 2026 Sep 9;21(9):e0356997. doi: 10.1371/journal.pone.0356997 (PMC13557361; doi:10.1371/journal.pone.0356997)
Supplement: Supplementary file 1 [file pone.0356997.s008.docx]

Dear Reviewers 1,2 and 3, thank you kindly for your comments and feedback. We have done our best to address your concerns and have described our changes (in black) in response to the comments (indented, in red):

**Responses to Reviewer # 1:**

The study shows clear variation in CAI across genomes, but the biological significance is not fully explained. The association with submitting institutions is interesting, yet it remains unclear whether this reflects true biological adaptation or potential biases (e.g., sampling, sequencing, or submission practices). This point needs further clarification.

All reviewers expressed similar concerns, so this result was de-emphasized.

The work is entirely computational and lacks functional validation. Without linking codon bias to gene expression, virulence, or fitness, the findings remain largely descriptive.

Analysis using a proteomics dataset was added to link codon bias to physiology. Virulence and fitness were not explored, which has been noted as a limitation in the revised manuscript.

The use of publicly available metadata may introduce bias. Institutional differences could reflect sequencing pipelines or clonal outbreaks rather than true biological variation. This limitation should be more clearly discussed.

This limitation has been expanded upon in the discussion section of the revised manuscript.

The interpretation of institutional clustering may be overstated, especially given sample imbalance and lack of control for clonality. Additional supporting analysis would strengthen this conclusion.

All reviewers expressed similar concerns, so this result was de-emphasized.

The Introduction should better justify the relevance of codon bias to Staphylococcus aureus biology.

This point has been expanded upon in the introduction of the revised manuscript.

Some results are overly descriptive and could be more concise.

Where possible, results have been presented in a more concise and less descriptive fashion. Additionally, statistical analyses were added in many places.

Figures need clearer labeling and interpretation.

Titles have been added to the figure to help with labeling, and the associated legends expanded.

Methods should more clearly define inclusion/exclusion criteria.

The methods section has been expanded and inclusion/exclusion criteria better defined.

**Responses to Reviewer # 2:**

1. Calculating codon specific values, e.g. CAI for non-coding sequences such as tRNAs are inappropriate. The codon bias analysis should be restricted to codon sequences; therefore, only protein-coding genes should be retained, and all other gene types should be filtered out from the downloaded dataset. Accordingly result section need to be updated.

We regret the error and have removed mention of tRNAs from the revised manuscript.

2. Most of the comparative analyses presented in the manuscript are primarily qualitative and are not supported by numerical data or statistical significance testing. For example, the difference in average CAI scores mentioned in the first paragraph of the Results section is not quantitatively substantiated within the text.

To address this, we added statistical analyses throughout. Additionally, linear mixed model analysis has been added to investigate differences between institutions.

3. It would be valuable to include biological interpretation of the differences in codon usage bias observed among strains obtained from different laboratories; numerical differences alone do not provide sufficient insight or merit.

To address this concern – which was shared by other reviewers - a proteomics dataset of *in vitro* protein expression in *Staphylococcus aureus* was analyzed*.* CAI was compared with protein expression for genes that overlapped. It was found that high CAI correlated with higher protein expression, although there was variation as to how well each gene mapped.

**Responses to Reviewer # 3:**

First, the authors do not sufficiently test for alternative methodological influences on the observed variation in CAI. Sequencing technology and amplification steps are known to induce GC bias in sequencing data. It is critical that the authors examine the role of sequencing method, assembly method, and other non-biological factors when examining closely related species.

To address this concern, the mean GC% of each contig was calculated and found to range between 0.331 and 0.338%. This is a modest range in GC content and unlikely to drive CAI scores. Additionally ,to control for potential differences between strains, CAI was determined with reference to each isolate’s 30S and 50S ribosomal genes.

Second, the authors do not sufficiently present their underlying data or methods in a way that would enable replication or ongoing analysis. Essential data that is missing from the supplemental data includes:
- The metadata associated with every sample analyzed. This should include the abbreviations, analyzed metadata, and any metrics generated during the analysis, such as the total number of genes annotated.
- All the genome annotations generated in this analysis.
- All of the raw CAI statistics for every gene
- Table S1 & S3– should report all the calculated loadings, not just the ones presented
- The CAI means, percentages, and interquartile ranges should all be reported.

>Information as to how metadata was processed has been added to the Methods section of the revised manuscript.

>A repository was created for the genome annotation results from the GAMMA queries along with CAI scores for each individual gene. Is it referenced in the text of the revised manuscript and available here: https://doi.org/10.5281/zenodo.20330626

>Tables S1 and S3 were removed because institution-related results were de-emphasized based on reviewer feedback.

>A new Table (S2) has been included to summarize all the CAI means and interquartile ranges.

Third, the methods lack details that should be included in the written description. While all of the code is available, the essential details should be included in the main text. This includes:
- The annotation step should include details such as whether protein or DNA sequences were used for blast, what metric was used to establish similarity, what the metric cutoff was, and how duplicate genes were handled.
- The general steps of the PCA and sPLS-DA, including how missing data was handled and any non-standard parameters

How PCA was run is included in the Methods section of the revised manuscript, including how missing data was handled. sPLS-DA analysis was removed.

Finally, I agree with the authors that a more compelling analysis would include additional codon bias metrics and analysis of tRNA diversity. There are codon usage metrics that are designed to identify the amount of selection acting on a genome overall, and on specific genes within a genome.

This would be a beneficial analysis. The way in which genes were queried meant only tRNAs that NCTC-8325 would have were included. A different approach that queried for genes using an alternative metric to find all available tRNAs would be needed. This is beyond the context of the paper, but its importance has been added to the Discussion of the revised manuscript so that hopefully this can be done in future studies.

The authors, at times, are clear that the variation observed cannot be linked to selection (line 266), but also freely speculate that there is a role for codon optimization in infection (Lines 276-277) or selection on codon usage for expression (Lines 291-293).

We don’t believe selection from the infection environment drives codon bias variation within genomes, however we do speculate that selective factors drive gene specific trends in CAI score. We amended the text throughout to better differentiate the two ideas.

Line 25-26 – “it must maximize.” The wording of this sentence anthropomorphizes the role of selection.

This has been adjusted

Results “Variation in average CAI between genomes” – the authors should report the amount of missing data in the genomes.

Table S1 has been added to show the number of genes considered for each genome.

Line 108 – the evidence for a misannotation of 50S ribosomal protein S15 should be reported.

This has been reported to GenBank.

Line 110 – “it is apparent that genes used in transcription…” The authors should conduct a statistical overrepresentation analysis to confirm the observation.

In this spot and other places, GSEA analysis has been added.

Line 120 – PCA – The PCA loadings would provide interesting information about the drivers of the separation seen on the PCA. If it is GC bias, this could provide additional interpretations.

The data fed into the PCA plot is the CAI scores of different genes; this has been clarified in the revised manuscript. GC bias was not a variable so it would not have been considered.

Line 225 – The analysis of the ribosomal genes starting on lines 225 would be better described first (before the other analyses). This was a lingering question I had while reading the manuscript and seeing the results. Addressing it after the fact is not as effective.

This has been moved forward in the Results section.

Line 278 – What “sub-Saharan genomes” are being referenced? Are these also S. aureus?

All *S. aureus* genomes; this has been in the text.

Line 318 – What does “dehydrated closed contig” mean?

This has been changed it to “sequences”.

Line 334 – Do the authors mean “dplyr” and not “deplyr”?

Corrected.
