# Supplementary material for: Codon bias variation in Staphylococcus aureus
Source: PLoS One. 2026 Sep 9;21(9):e0356997. doi: 10.1371/journal.pone.0356997 (PMC13557361; doi:10.1371/journal.pone.0356997)
Supplement: Supplementary file 2 [file pone.0356997.s009.docx]

We would like to thank reviewer # 3 for their comments. We have incorporated their feedback. A summary of the changes we made in response to each comment is below:

Reviewer #3: The authors of “Codon Bias Variation in Staphylococcus aureus” have made substantial changes to the manuscript to address the comments of the reviewers.
I was unable to assess Figure 5 as it was not included in the submission

We apologize for this omission; Figure 5 is now included.

There are remaining issues to be addressed prior to publication.
Line 28-29: Fitness may increase during replication, but that depends on the relative forces of drift and selection.

‘Replication’ has been changed to “adaptation”.

Line 50-51: There is some evidence that codon bias acts during transcription and is not exclusively post-transcriptional. I’m not sure how it can control protein product after transcription but before translation.

This statement has been removed.

Line 53: A gene with high CAI is hypothesized to be translated at a high rate

The term “high” has been added.

Line 54: “may not be translated” – this phrase is used throughout. What biologically does this mean? Are you referring to pseudogenes, RNAs, etc?

This could refer to any gene that is not translated – pseudogenes or RNAs.

Fig1A: Based on the scale it is hard to see how the high and low genomes differ. It would be good to report the mean of the mean CAIs for the top and bottom 10. Are the genomes sorted in any way in Fig1A and B? It may be useful to sort them by mean.

They are sorted by mean; means for each genome have been added to Table S1.

Line 111-113 – When comparing the number of genes within each institution, is this the total number of genes regardless of the number of genomes? It is difficult to understand how these absolute values compare without knowing what percent they are. This is purely observational, a statistical test could be done on the count data to support the conclusions drawn.

We now report the number of high- and low-CAI genes as a percentage of the total number of genes assessed per institution, rather than as raw counts, to allow for direct comparisons across institutions with different numbers of submitted genomes. We have also added statistical analyses of these comparisons. We updated the Methods section based on the new analyses performed.

Line 125-126: Is the proportional number of ribosomal proteins with high CAI scores reported anywhere? Are they statistically indistinguishable or observationally similar?

We now explicitly report the percentage of high-CAI genes that are ribosomal proteins for each institution, and have added statistical testing to address whether these proportions differ. A test of equality of proportions across all institutions showed no significant difference; a Fisher's exact test comparing AKUHN against the pooled other institutions also showed no difference.

Figure 3B: Why are there two data points associated with “metal ion binding”?

There are multiple sub GO groups that relate the metal ion binding so there is more than one point.

Line 188: The subheadings in this section are confusing.

Subheadings have been removed.

Figure 4G-I: These are not cited in the manuscript.

Figures are now cited in the text.

Line 252: The correlation referred to in this section, is that a slope or an R2?

This is the correlation value “r” outputted by the R Studio cor.test function. We did not fit a linear model so there is no slope to report.

Line 299: It is unsurprising that the lowest CAI would be in genes that are hypothetical and therefore less likely to be true protein coding genes. It would be more biologically relevant to present the lowest CAI of non-hypothetical proteins.

We agree that it would be interesting to report the high and low CAI genes and their functions. However, we feel that the GSEA analysis does a better job of presenting the biological importance of up/down genes rather than presenting specific examples.

Line 361: When the authors exclude likely non-aureus isolates, is this done only with the annotation number or were other methods employed to remove non-aureus isolates?

A single non-*S. aureus* isolate was removed. We used its reference number to find and remove it. This was based on the low number of *S. aureus* genes it had.

Line 433: What type/method of p-value adjustment (multiple testing correction) was done?

In the original version, no adjustment was used. We have now amended our code to use **BH (Benjamini-Hochberg). The figures reflect that change.**

Linear mixed modeling methods are not included in the methods section.

This has been added

Line 475: Why was mean CAI across the genomes used for the comparison to proteomics? Would you expect that all genomes would fit the proteomics data equally well?

Mean CAI was not used; the CAI from LAC, the same strain used for proteomics was used. This is described here: 251-253 lines
