# Supplementary material for: Codon bias variation in Staphylococcus aureus
Source: PLoS One. 2026 Sep 9;21(9):e0356997. doi: 10.1371/journal.pone.0356997 (PMC13557361; doi:10.1371/journal.pone.0356997)
Supplement: Peer review history

# Peer review: Decision Letter 0

**DOI:** 10.1371/journal.pone.0356997.r001

**Authors:** Jordy Evan Sulaiman

30 Apr 2026

Codon Bias Variation in Staphylococcus aureus

PLOS One

Dear Dr. Patel,

Thank you for submitting your manuscript to PLOS ONE. After careful consideration, we feel that it has merit but does not fully meet PLOS ONE’s publication criteria as it currently stands. Therefore, we invite you to submit a revised version of the manuscript that addresses the points raised during the review process.

Please submit your revised manuscript by Jun 13 2026 11:59PM. If you will need more time than this to complete your revisions, please reply to this message or contact the journal office at plosone@plos.org. When you're ready to submit your revision, log on to https://www.editorialmanager.com/pone/ and select the 'Submissions Needing Revision' folder to locate your manuscript file.

Please include the following items when submitting your revised manuscript:

- A letter that responds to each point raised by the academic editor and reviewer(s). You should upload this letter as a separate file labeled 'Response to Reviewers'.
- A marked-up copy of your manuscript that highlights changes made to the original version. You should upload this as a separate file labeled 'Revised Manuscript with Track Changes'.
- An unmarked version of your revised paper without tracked changes. You should upload this as a separate file labeled 'Manuscript'.

If you would like to make changes to your financial disclosure, please include your updated statement in your cover letter. Guidelines for resubmitting your figure files are available below the reviewer comments at the end of this letter.

If applicable, we recommend that you deposit your laboratory protocols in protocols.io to enhance the reproducibility of your results. Protocols.io assigns your protocol its own identifier (DOI) so that it can be cited independently in the future. For instructions see: https://journals.plos.org/plosone/s/submission-guidelines#loc-laboratory-protocols. Additionally, PLOS ONE offers an option for publishing peer-reviewed Lab Protocol articles, which describe protocols hosted on protocols.io. Read more information on sharing protocols at https://plos.org/protocols?utm_medium=editorial-email&utm_source=authorletters&utm_campaign=protocols.

As the corresponding author, your ORCID iD is verified in the submission system and will appear in the published article. PLOS supports the use of ORCID, and we encourage all coauthors to register for an ORCID iD and use it as well. Please encourage your coauthors to verify their ORCID iD within the submission system before final acceptance, as unverified ORCID iDs will not appear in the published article. *Only*  the individual author can complete the verification step; PLOS staff *cannot*  verify ORCID iDs on behalf of authors.

We look forward to receiving your revised manuscript.

Kind regards,

Jordy Evan Sulaiman, Ph.D.

Academic Editor

PLOS One

Journal Requirements:

When submitting your revision, we need you to address these additional requirements.

1.Please ensure that your manuscript meets PLOS ONE’s style requirements, including those for file naming. The PLOS ONE style templates can be found at

https://journals.plos.org/plosone/s/file?id=wjVg/PLOSOne_formatting_sample_main_body.pdf and

https://journals.plos.org/plosone/s/file?id=ba62/PLOSOne_formatting_sample_title_authors_affiliations.pdf

2. Please note that funding information should not appear in any section or other areas of your manuscript. We will only publish funding information present in the Funding Statement section of the online submission form. Please remove any funding-related text from the manuscript.

3. Thank you for stating the following financial disclosure:

“Research reported in this publication was supported by the National Institute of Arthritis and Musculoskeletal and Skin Diseases of the National Institute of Health under award number T32 AR056950.”

Please state what role the funders took in the study.  If the funders had no role, please state: 'The funders had no role in study design, data collection and analysis, decision to publish, or preparation of the manuscript.'

If this statement is not correct you must amend it as needed.

Please include this amended Role of Funder statement in your cover letter; we will change the online submission form on your behalf.

4. Thank you for stating the following in the Competing Interests section:

“R.P. reports grants from MicuRx Pharmaceuticals and ImmuCell. R.P. is a consultant to PhAST, Iterum Therapeutics, DEEPULL DIAGNOSTICS, Claryx, Inc., Nostics, and CARB-X. In addition, R.P. has a patent on Bordetella pertussis/parapertussis PCR issued, a patent on a device/method for sonication, a patent on PET imaging of bacterial infection with a PET probe filed, and a patent on an anti-biofilm substance issued. R.P. receives honoraria from Up-to-Date and the Infectious Diseases Board Review Course. The other authors have no conflicts of interest to declare.”

We note that one or more of the authors are employed by a commercial company: MicuRx Pharmaceuticals. ImmuCell, PhAST, Iterum Therapeutics, DEEPULL DIAGNOSTICS, Claryx, Inc., Nostics, and CARB-X.

1. Please provide an amended Funding Statement declaring this commercial affiliation, as well as a statement regarding the Role of Funders in your study. If the funding organization did not play a role in the study design, data collection and analysis, decision to publish, or preparation of the manuscript and only provided financial support in the form of authors' salaries and/or research materials, please review your statements relating to the author contributions, and ensure you have specifically and accurately indicated the role(s) that these authors had in your study. You can update author roles in the Author Contributions section of the online submission form.

Please also include the following statement within your amended Funding Statement.

“The funder provided support in the form of salaries for authors [insert relevant initials], but did not have any additional role in the study design, data collection and analysis, decision to publish, or preparation of the manuscript. The specific roles of these authors are articulated in the ‘author contributions’ section.”

If your commercial affiliation did play a role in your study, please state and explain this role within your updated Funding Statement.

2. Please also provide an updated Competing Interests Statement declaring this commercial affiliation along with any other relevant declarations relating to employment, consultancy, patents, products in development, or marketed products, etc.

Within your Competing Interests Statement, please confirm that this commercial affiliation does not alter your adherence to all PLOS ONE policies on sharing data and materials by including the following statement: 'This does not alter our adherence to  PLOS ONE policies on sharing data and materials.” (as detailed online in our guide for authors http://journals.plos.org/plosone/s/competing-interests) . If this adherence statement is not accurate and  there are restrictions on sharing of data and/or materials, please state these. Please note that we cannot proceed with consideration of your article until this information has been declared.

Please include both an updated Funding Statement and Competing Interests Statement in your cover letter. We will change the online submission form on your behalf.

5. We note that you have a patent relating to material pertinent to this article. Please provide an amended statement of Competing Interests to declare this patent (with details including name and number), along with any other relevant declarations relating to employment, consultancy, patents, products in development or modified products etc. Please confirm that this does not alter your adherence to all PLOS ONE policies on sharing data and materials, as detailed online in our guide for authors http://journals.plos.org/plosone/s/competing-interests by including the following statement: 'This does not alter our adherence to  PLOS ONE policies on sharing data and materials.” If there are restrictions on sharing of data and/or materials, please state these. Please note that we cannot proceed with consideration of your article until this information has been declared.

This information should be included in your cover letter; we will change the online submission form on your behalf.

6. Your ethics statement should only appear in the Methods section of your manuscript. If your ethics statement is written in any section besides the Methods, please move it to the Methods section and delete it from any other section. Please ensure that your ethics statement is included in your manuscript, as the ethics statement entered into the online submission form will not be published alongside your manuscript.

7. Please note that your Data Availability Statement is currently missing the repository name and/or the DOI/accession number of each dataset OR a direct link to access each database. If your manuscript is accepted for publication, you will be asked to provide these details on a very short timeline. We therefore suggest that you provide this information now, though we will not hold up the peer review process if you are unable.

8. If the reviewer comments include a recommendation to cite specific previously published works, please review and evaluate these publications to determine whether they are relevant and should be cited. There is no requirement to cite these works unless the editor has indicated otherwise.

Additional Editor Comments:

I have now received the reviewers' comments. While the reviewers and I think that the work is interesting and could be valuable to the community, some issues need to be addressed and clarified before the manuscript can be considered further. Therefore, we invite you to submit a revised version of the manuscript that addresses the points raised during the review process. In addition to addressing specific reviewer comments, please pay attention to the statistical analyses used in the manuscript and be reminded to make all data in the paper publicly available.

[Note: HTML markup is below. Please do not edit.]

Reviewers' comments:

Reviewer’s Responses to Questions

**Comments to the Author**

1. Is the manuscript technically sound, and do the data support the conclusions?

Reviewer #1: Yes

Reviewer #2: Partly

Reviewer #3: Partly

**********

2. Has the statistical analysis been performed appropriately and rigorously?

Reviewer #1: I Don't Know

Reviewer #2: No

Reviewer #3: No

**********

3. Have the authors made all data underlying the findings in their manuscript fully available?

The PLOS Data policy

Reviewer #1: Yes

Reviewer #2: Yes

Reviewer #3: No

**********

4. Is the manuscript presented in an intelligible fashion and written in standard English?

Reviewer #1: Yes

Reviewer #2: Yes

Reviewer #3: Yes

**********

Reviewer #1: Greetings

Goid work. But edit the points below:

Major Comments

The study shows clear variation in CAI across genomes, but the biological significance is not fully explained. The association with submitting institutions is interesting, yet it remains unclear whether this reflects true biological adaptation or potential biases (e.g., sampling, sequencing, or submission practices). This point needs further clarification.

The work is entirely computational and lacks functional validation. Without linking codon bias to gene expression, virulence, or fitness, the findings remain largely descriptive.

The use of publicly available metadata may introduce bias. Institutional differences could reflect sequencing pipelines or clonal outbreaks rather than true biological variation. This limitation should be more clearly discussed.

The interpretation of institutional clustering may be overstated, especially given sample imbalance and lack of control for clonality. Additional supporting analysis would strengthen this conclusion.

Minor Comments

The Introduction should better justify the relevance of codon bias to Staphylococcus aureus biology.

Some results are overly descriptive and could be more concise.

Figures need clearer labeling and interpretation.

Methods should more clearly define inclusion/exclusion criteria.

Kind regards.

Reviewer #2: This study examines codon usage bias (CUB) in the human pathogen Staphylococcus aureus through genome-wide computational analyses using publicly available data. It explores variations both within and among strains derived from diverse sources. Overall, the work offers valuable insights into the evolutionary and functional significance of codon usage patterns in this clinically important bacterium. Given its relevance, I recommend this manuscript for publication, subject to revisions addressing the following points:

1. Calculating codon specific values, e.g. CAI for non-coding sequences such as tRNAs are inappropriate. The codon bias analysis should be restricted to codon sequences; therefore, only protein-coding genes should be retained, and all other gene types should be filtered out from the downloaded dataset. Accordingly result section need to be updated.

2. Most of the comparative analyses presented in the manuscript are primarily qualitative and are not supported by numerical data or statistical significance testing. For example, the difference in average CAI scores mentioned in the first paragraph of the Results section is not quantitatively substantiated within the text.

3. It would be valuable to include biological interpretation of the differences in codon usage bias observed among strains obtained from different laboratories; numerical differences alone do not provide sufficient insight or merit.

Reviewer #3: The authors of Codon Bias Variation in Staphylococcus aureus present a descriptive analysis of the variation in codon usage bias of many samples collected across geographic regions and clinical settings. They identify differences in codon usage, as measured by CAI, associated with samples from one institution.

I have major concerns with the manuscript that the authors must address prior to publication to ensure scientific rigor.

First, the authors do not sufficiently test for alternative methodological influences on the observed variation in CAI. Sequencing technology and amplification steps are known to induce GC bias in sequencing data. It is critical that the authors examine the role of sequencing method, assembly method, and other non-biological factors when examining closely related species.

Second, the authors do not sufficiently present their underlying data or methods in a way that would enable replication or ongoing analysis. Essential data that is missing from the supplemental data includes:

- The metadata associated with every sample analyzed. This should include the abbreviations, analyzed metadata, and any metrics generated during the analysis, such as the total number of genes annotated.

- All the genome annotations generated in this analysis.

- All of the raw CAI statistics for every gene

- Table S1 & S3– should report all the calculated loadings, not just the ones presented

- The CAI means, percentages, and interquartile ranges should all be reported.

Third, the methods lack details that should be included in the written description. While all of the code is available, the essential details should be included in the main text. This includes:

- The annotation step should include details such as whether protein or DNA sequences were used for blast, what metric was used to establish similarity, what the metric cutoff was, and how duplicate genes were handled.

- The general steps of the PCA and sPLS-DA, including how missing data was handled and any non-standard parameters

Finally, I agree with the authors that a more compelling analysis would include additional codon bias metrics and analysis of tRNA diversity. There are codon usage metrics that are designed to identify the amount of selection acting on a genome overall, and on specific genes within a genome. The authors, at times, are clear that the variation observed cannot be linked to selection (line 266), but also freely speculate that there is a role for codon optimization in infection (Lines 276-277) or selection on codon usage for expression (Lines 291-293).

There are other minor concerns the authors should address

Line 25-26 – “it must maximize.” The wording of this sentence anthropomorphizes the role of selection.

Results “Variation in average CAI between genomes” – the authors should report the amount of missing data in the genomes.

Line 108 – the evidence for a misannotation of 50S ribosomal protein S15 should be reported.

Line 110 – “it is apparent that genes used in transcription…” The authors should conduct a statistical overrepresentation analysis to confirm the observation.

Line 120 – PCA – The PCA loadings would provide interesting information about the drivers of the separation seen on the PCA. If it is GC bias, this could provide additional interpretations.

Line 225 – The analysis of the ribosomal genes starting on lines 225 would be better described first (before the other analyses). This was a lingering question I had while reading the manuscript and seeing the results. Addressing it after the fact is not as effective.

Line 278 – What “sub-Saharan genomes” are being referenced? Are these also S. aureus?

Line 318 – What does “dehydrated closed contig” mean?

Line 334 – Do the authors mean “dplyr” and not “deplyr”?

**********

what does this mean?). If published, this will include your full peer review and any attached files.

If you choose “no”, your identity will remain anonymous but your review may still be made public.

**Do you want your identity to be public for this peer review?**  For information about this choice, including consent withdrawal, please see our Privacy Policy

Reviewer #1: No

Reviewer #2: No

Reviewer #3: No

**********

[NOTE: If reviewer comments were submitted as an attachment file, they will be attached to this email and accessible via the submission site. Please log into your account, locate the manuscript record, and check for the action link "View Attachments". If this link does not appear, there are no attachment files.]

To ensure your figures meet our technical requirements, please review our figure guidelines: https://journals.plos.org/plosone/s/figures

You may also use PLOS’s free figure tool, NAAS, to help you prepare publication quality figures: https://journals.plos.org/plosone/s/figures#loc-tools-for-figure-preparation.

NAAS will assess whether your figures meet our technical requirements by comparing each figure against our figure specifications.

# Author comment: Author response to Decision Letter 1

**DOI:** 10.1371/journal.pone.0356997.r002

12 Jun 2026

See attachment

Attachment Submitted filename: ResponsestoReviewer.docx

# Peer review: Decision Letter 1

**DOI:** 10.1371/journal.pone.0356997.r003

**Authors:** Jordy Evan Sulaiman

30 Jun 2026

Dear Dr. Patel,

Thank you for submitting your manuscript to PLOS ONE. After careful consideration, we feel that it has merit but does not fully meet PLOS ONE’s publication criteria as it currently stands. Therefore, we invite you to submit a revised version of the manuscript that addresses the points raised during the review process.

==============================

Dear Dr. Patel,

Thank you for submitting your revised manuscript, which has now been reviewed by the original reviewers. While the reviewers think that most of the comments have been adequately addressed, there are still several lingering issues that need to be addressed before we can consider the manuscript further. Please respond to the reviewers' comments and submit a point-by-point response, together with the revised manuscript. We look forward to receiving your revisions!

==============================

Please submit your revised manuscript by Aug 14 2026 11:59PM. If you will need more time than this to complete your revisions, please reply to this message or contact the journal office at plosone@plos.org. When you're ready to submit your revision, log on to https://www.editorialmanager.com/pone/ and select the 'Submissions Needing Revision' folder to locate your manuscript file.

- A letter that responds to each point raised by the academic editor and reviewer(s). You should upload this letter as a separate file labeled 'Response to Reviewers'.
- A marked-up copy of your manuscript that highlights changes made to the original version. You should upload this as a separate file labeled 'Revised Manuscript with Track Changes'.
- An unmarked version of your revised paper without tracked changes. You should upload this as a separate file labeled 'Manuscript'.

If you would like to make changes to your financial disclosure, please include your updated statement in your cover letter. Guidelines for resubmitting your figure files are available below the reviewer comments at the end of this letter.

If applicable, we recommend that you deposit your laboratory protocols in protocols.io to enhance the reproducibility of your results. Protocols.io assigns your protocol its own identifier (DOI) so that it can be cited independently in the future. For instructions see: https://journals.plos.org/plosone/s/submission-guidelines#loc-laboratory-protocols. Additionally, PLOS ONE offers an option for publishing peer-reviewed Lab Protocol articles, which describe protocols hosted on protocols.io. Read more information on sharing protocols at https://plos.org/protocols?utm_medium=editorial-email&utm_source=authorletters&utm_campaign=protocols.

As the corresponding author, your ORCID iD is verified in the submission system and will appear in the published article. PLOS supports the use of ORCID, and we encourage all coauthors to register for an ORCID iD and use it as well. Please encourage your coauthors to verify their ORCID iD within the submission system before final acceptance, as unverified ORCID iDs will not appear in the published article. *Only* the individual author can complete the verification step; PLOS staff *cannot* verify ORCID iDs on behalf of authors.

We look forward to receiving your revised manuscript.

Kind regards,

Jordy Evan Sulaiman, Ph.D.

Academic Editor

PLOS One

Journal Requirements:

If the reviewer comments include a recommendation to cite specific previously published works, please review and evaluate these publications to determine whether they are relevant and should be cited. There is no requirement to cite these works unless the editor has indicated otherwise.

Please review your reference list to ensure that it is complete and correct. If you have cited papers that have been retracted, please include the rationale for doing so in the manuscript text, or remove these references and replace them with relevant current references. Any changes to the reference list should be mentioned in the rebuttal letter that accompanies your revised manuscript. If you need to cite a retracted article, indicate the article’s retracted status in the References list and also include a citation and full reference for the retraction notice.

Additional Editor Comments:

Dear Dr. Patel,

Thank you for submitting your revised manuscript, which has now been reviewed by the original reviewers. While the reviewers think that most of the comments have been adequately addressed, there are still several lingering issues that need to be addressed before we can consider the manuscript further. Please respond to the reviewers' comments and submit a point-by-point response, together with the revised manuscript. We look forward to receiving your revisions!

Best regards,

Jordy

[Note: HTML markup is below. Please do not edit.]

Reviewers' comments:

Reviewer’s Responses to Questions

**Comments to the Author**

Reviewer #1: All comments have been addressed

Reviewer #2: All comments have been addressed

Reviewer #3: (No Response)

**********

2. Is the manuscript technically sound, and do the data support the conclusions?

Reviewer #1: (No Response)

Reviewer #2: Yes

Reviewer #3: Yes

**********

3. Has the statistical analysis been performed appropriately and rigorously?

Reviewer #1: Yes

Reviewer #2: Yes

Reviewer #3: No

**********

4. Have the authors made all data underlying the findings in their manuscript fully available?

The PLOS Data policy

Reviewer #1: Yes

Reviewer #2: Yes

Reviewer #3: Yes

**********

5. Is the manuscript presented in an intelligible fashion and written in standard English?

Reviewer #1: Yes

Reviewer #2: Yes

Reviewer #3: Yes

**********

Reviewer #1: (No Response)

Reviewer #2: (No Response)

Reviewer #3: The authors of “Codon Bias Variation in Staphylococcus aureus” have made substantial changes to the manuscript to address the comments of the reviewers.

I was unable to assess Figure 5 as it was not included in the submission.

There are remaining issues to be addressed prior to publication.

Line 28-29: Fitness may increase during replication, but that depends on the relative forces of drift and selection.

Line 50-51: There is some evidence that codon bias acts during transcription and is not exclusively post-transcriptional. I’m not sure how it can control protein product after transcription but before translation.

Line 53: A gene with high CAI is hypothesized to be translated at a high rate

Line 54: “may not be translated” – this phrase is used throughout. What biologically does this mean? Are you referring to pseudogenes, RNAs, etc?

Fig1A: Based on the scale it is hard to see how the high and low genomes differ. It would be good to report the mean of the mean CAIs for the top and bottom 10. Are the genomes sorted in any way in Fig1A and B? It may be useful to sort them by mean.

Line 111-113 – When comparing the number of genes within each institution, is this the total number of genes regardless of the number of genomes? It is difficult to understand how these absolute values compare without knowing what percent they are. This is purely observational, a statistical test could be done on the count data to support the conclusions drawn.

Line 125-126: Is the proportional number of ribosomal proteins with high CAI scores reported anywhere? Are they statistically indistinguishable or observationally similar?

Figure 3B: Why are there two data points associated with “metal ion binding”?

Line 188: The subheadings in this section are confusing.

Figure 4G-I: These are not cited in the manuscript.

Line 252: The correlation referred to in this section, is that a slope or an R2?

Line 299: It is unsurprising that the lowest CAI would be in genes that are hypothetical and therefore less likely to be true protein coding genes. It would be more biologically relevant to present the lowest CAI of non-hypothetical proteins.

Line 361: When the authors exclude likely non-aureus isolates, is this done only with the annotation number or were other methods employed to remove non-aureus isolates?

Line 433: What type/method of p-value adjustment (multiple testing correction) was done?

Linear mixed modeling methods are not included in the methods section.

Line 475: Why was mean CAI across the genomes used for the comparison to proteomics? Would you expect that all genomes would fit the proteomics data equally well?

**********

what does this mean?). If published, this will include your full peer review and any attached files.

If you choose “no”, your identity will remain anonymous but your review may still be made public.

**Do you want your identity to be public for this peer review?** For information about this choice, including consent withdrawal, please see our Privacy Policy

Reviewer #1: No

Reviewer #2: No

Reviewer #3: No

**********

[NOTE: If reviewer comments were submitted as an attachment file, they will be attached to this email and accessible via the submission site. Please log into your account, locate the manuscript record, and check for the action link "View Attachments". If this link does not appear, there are no attachment files.]

To ensure your figures meet our technical requirements, please review our figure guidelines: https://journals.plos.org/plosone/s/figures

You may also use PLOS’s free figure tool, NAAS, to help you prepare publication quality figures: https://journals.plos.org/plosone/s/figures#loc-tools-for-figure-preparation.

NAAS will assess whether your figures meet our technical requirements by comparing each figure against our figure specifications.

# Author comment: Author response to Decision Letter 2

**DOI:** 10.1371/journal.pone.0356997.r004

10 Aug 2026

See attachment

Attachment Submitted filename: ReviewerResponse.docx

# Editor report: Decision Letter 2

**DOI:** 10.1371/journal.pone.0356997.r005

**Authors:** Jordy Evan Sulaiman

12 Aug 2026

Codon Bias Variation in Staphylococcus aureus

PONE-D-26-16137R2

Dear Dr. Patel,

We’re pleased to inform you that your manuscript has been judged scientifically suitable for publication and will be formally accepted for publication once it meets all outstanding technical requirements.

Within one week, you’ll receive an e-mail detailing the required amendments. When these have been addressed, you’ll receive a formal acceptance letter and your manuscript will be scheduled for publication.

An invoice will be generated when your article is formally accepted. Please note, if your institution has a publishing partnership with PLOS and your article meets the relevant criteria, all or part of your publication costs will be covered. Please make sure your user information is up-to-date by logging into Editorial Manager at Editorial Manager® and clicking the ‘Update My Information' link at the top of the page. For questions related to billing, please contact billing support.

If your institution or institutions have a press office, please notify them about your upcoming paper to help maximize its impact. If they’ll be preparing press materials, please inform our press team as soon as possible -- no later than 48 hours after receiving the formal acceptance. Your manuscript will remain under strict press embargo until 2 pm Eastern Time on the date of publication. For more information, please contact onepress@plos.org.

Kind regards,

Jordy Evan Sulaiman, Ph.D.

Academic Editor

PLOS One

Additional Editor Comments (optional):

Dear Dr. Patel,

Thank you for submitting the revised manuscript, which is now suitable for publication in PLOS One. Once again, thank you for your patience and efforts throughout the review process.

Reviewers' comments:

# Editor report: Acceptance letter

**DOI:** 10.1371/journal.pone.0356997.r006

**Authors:** Jordy Evan Sulaiman

PONE-D-26-16137R2

PLOS One

Dear Dr. Patel,

I'm pleased to inform you that your manuscript has been deemed suitable for publication in PLOS One. Congratulations! Your manuscript is now being handed over to our production team.

At this stage, our production department will prepare your paper for publication. This includes ensuring the following:

* All references, tables, and figures are properly cited

* All relevant supporting information is included in the manuscript submission,

* There are no issues that prevent the paper from being properly typeset

You will receive further instructions from the production team, including instructions on how to review your proof when it is ready. Please keep in mind that we are working through a large volume of accepted articles, so please give us a few days to review your paper and let you know the next and final steps.

Lastly, if your institution or institutions have a press office, please let them know about your upcoming paper now to help maximize its impact. If they'll be preparing press materials, please inform our press team within the next 48 hours. Your manuscript will remain under strict press embargo until 2 pm Eastern Time on the date of publication. For more information, please contact onepress@plos.org.

You will receive an invoice from PLOS for your publication fee after your manuscript has reached the completed accept phase. If you receive an email requesting payment before acceptance or for any other service, this may be a phishing scheme. Learn how to identify phishing emails and protect your accounts at https://explore.plos.org/phishing.

If we can help with anything else, please email us at customercare@plos.org.

Thank you for submitting your work to PLOS One and supporting open access.

Kind regards,

PLOS One Editorial Office Staff

on behalf of

Dr. Jordy Evan Sulaiman

Academic Editor

PLOS One
